# Molecular dynamics and mutational analysis of the catalytic and translocation cycle of RNA polymerase

**DOI:** 10.1186/2046-1682-5-11

**Published:** 2012-06-07

**Authors:** Maria L Kireeva, Kristopher Opron, Steve A Seibold, Céline Domecq, Robert I Cukier, Benoit Coulombe, Mikhail Kashlev, Zachary F Burton

**Affiliations:** 1 Gene Regulation and Chromosome Biology Laboratory, National Cancer Institute, Frederick, MD, 21702-1201, USA; 2 Department of Biochemistry and Molecular Biology, Michigan State University, E. Lansing, MI, 48824-1319, USA; 3 Department of Chemistry, Michigan State University, E. Lansing, MI, 48824, USA; 4 Department of Chemistry, University of Saint Mary, Leavenworth, KS, 66048, USA; 5 Gene Transcription and Proteomics Laboratory, Institut de Recherches Cliniques de Montréal (IRCM), 110, Avenue des Pins Ouest, Montréal, Québec, H2W 1R7, CANADA; 6 Department of Biochemistry, Université de Montréal, Montréal, Québec, H3C 3J7, CANADA

## Abstract

**Background:**

During elongation, multi-subunit RNA polymerases (RNAPs) cycle between phosphodiester bond formation and nucleic acid translocation. In the conformation associated with catalysis, the mobile “trigger loop” of the catalytic subunit closes on the nucleoside triphosphate (NTP) substrate. Closing of the trigger loop is expected to exclude water from the active site, and dehydration may contribute to catalysis and fidelity. In the absence of a NTP substrate in the active site, the trigger loop opens, which may enable translocation. Another notable structural element of the RNAP catalytic center is the “bridge helix” that separates the active site from downstream DNA. The bridge helix may participate in translocation by bending against the RNA/DNA hybrid to induce RNAP forward movement and to vacate the active site for the next NTP loading. The transition between catalytic and translocation conformations of RNAP is not evident from static crystallographic snapshots in which macromolecular motions may be restrained by crystal packing.

**Results:**

All atom molecular dynamics simulations of *Thermus thermophilus* (Tt) RNAP reveal flexible hinges, located within the two helices at the base of the trigger loop, and two glycine hinges clustered near the N-terminal end of the bridge helix. As simulation progresses, these hinges adopt distinct conformations in the closed and open trigger loop structures. A number of residues (described as “switch” residues) trade atomic contacts (ion pairs or hydrogen bonds) in response to changes in hinge orientation. In vivo phenotypes and in vitro activities rendered by mutations in the hinge and switch residues in *Saccharomyces cerevisiae* (Sc) RNAP II support the importance of conformational changes predicted from simulations in catalysis and translocation. During simulation, the elongation complex with an open trigger loop spontaneously translocates forward relative to the elongation complex with a closed trigger loop.

**Conclusions:**

Switching between catalytic and translocating RNAP forms involves closing and opening of the trigger loop and long-range conformational changes in the atomic contacts of amino acid side chains, some located at a considerable distance from the trigger loop and active site. Trigger loop closing appears to support chemistry and the fidelity of RNA synthesis. Trigger loop opening and limited bridge helix bending appears to promote forward nucleic acid translocation.

## Background

In each phosphodiester bond addition cycle during RNA elongation, there are multiple intermediates, but two major events are catalysis (phosphodiester bond formation) and translocation (movement of nucleic acids). A major question in transcription, therefore, is how RNA polymerase (RNAP) conformations and dynamics support cycling between catalysis and translocation. From structures, it appears that a closed conformation of the “trigger loop” supports catalysis [1,2]. Two models have been put forward for translocation. In one model, incoming NTP substrates act as allosteric effectors driving forward translocation [3-7]. In another model, thermally driven oscillations of RNAP elements stimulate nucleic acids to slide forward [8-11]. In this work, translocation is modeled and analyzed mostly in the presence of a NTP substrate, which may influence nucleic acid movements through RNAP.

Two defining features of the multi-subunit RNAP ternary elongation complex (TEC) are the “bridge helix” and the “trigger loop”, which are implicated in translocation, nucleotide sequestration and fidelity of NTP incorporation [2,3,9,11]. The bridge helix, which abuts the active site and contacts the leading edge of the RNA/DNA hybrid, may function as a ratchet to drive forward translocation, for instance, by bending against the RNA/DNA hybrid [12]. *Thermus thermophilus* (Tt) RNAP bridge helix (β’ 1067 to 1104) may have a glycine hinge (or two neighboring hinges) within the segment 1076-GARKGG-1081 [2,13-15]. Recent high throughput bridge helix mutagenesis of archaeal *Methanocaldococcus jannashii* (Mj) RNAP combined with molecular dynamics simulation supports the presence of two major bend points, centered at Mj RNAP 809-GG-810 (corresponding to Tt RNAP 1076-GA-1077) and G825 (Tt RNAP G1092) [14,15]. In a Tt RNAP TEC with a closed trigger loop, molecular dynamics simulations also support a primary bend near 1080-GG-1081 [13].

In the active site of the Tt RNAP TEC, the flexible trigger loop (β’ 1219 to 1265) adopts closed and open conformations [1,16]. The RNAP TEC with a closed trigger loop has been observed in the presence of an accurately loaded NTP substrate and is thought to be the catalytic conformation. The open trigger loop TEC, thought to be the translocating form [17,18], was observed in the absence of a NTP substrate or in the presence of the antibiotic streptolydigin, which may inhibit transcription by allosterically blocking trigger loop closing, NTP substrate alignment or both. The RNAP trigger loop may have multiple roles in fidelity: maintaining the accuracy of RNA synthesis and maintaining the precision of the single base step length. Additionally, closing the trigger loop may dehydrate the RNAP catalytic center contributing to the fidelity of NTP recognition [13]. Within α-helical segments surrounding the trigger loop, Tt RNAP has been suggested to have two GXP hinges [2,14,19], 1230-GEP-1232 and 1255-GLP-1257 [20]. It is not clear whether these hinges are involved in translocation and/or phosphodiester bond formation or how motion of these hinges might be coupled to more distant conformational changes of RNAP.

We addressed the effects of trigger loop closing and opening on the RNAP TEC conformation using all atom molecular dynamics simulations, starting from known crystal structures (PDB files 2PPB, 2O5I and 2O5J) [16,21]. Molecular dynamics is a method to refine x-ray crystal structures in the presence of water and counterions and in the absence of crystal packing forces [22-24]. Additionally, molecular dynamics can explore otherwise unknown TEC conformations, and probe potential interactions between atoms. Recently, all atom molecular dynamics simulations of *Saccharomyces cerevisiae* (Sc) RNAP II were used to investigate trigger loop closing, NTP binding and pyrophosphate release [25,26]. For the current studies, Tt RNAP TECs were chosen over Sc RNAP II TECs for molecular dynamics analyses because of the higher resolution, better catalytic geometry and enhanced active site closing in the 2O5J Tt RNAP closed trigger loop TEC x-ray structure [16] compared to the 2E2H Sc RNAP II structure [1]. Tighter active site closing is expected to enhance the contrast between the closed and open Tt RNAP TECs in simulations.

Modeling of Tt RNAP TECs with accurately loaded ATP substrates established that, as molecular dynamics simulations progress, closed and open TECs diverge into two distinct conformations that include bending of the bridge helix and trigger loop hinges and switching of multiple atomic contacts between amino acid residues that can be distal from the trigger loop and from the active site. The functional importance of hinge movements and the long-range conformational changes identified in the simulations were supported by site-directed mutagenesis and in vitro characterization of mutant RNAPs. Conformational changes during cycling between catalytic and translocating forms of RNAP indicate that closing and opening of the trigger loop and limited bridge helix bending may check and maintain the accuracy of translocation steps.

## Results and discussion

### Molecular dynamics simulations of closed and open RNAP TECs

**Figure 1:**
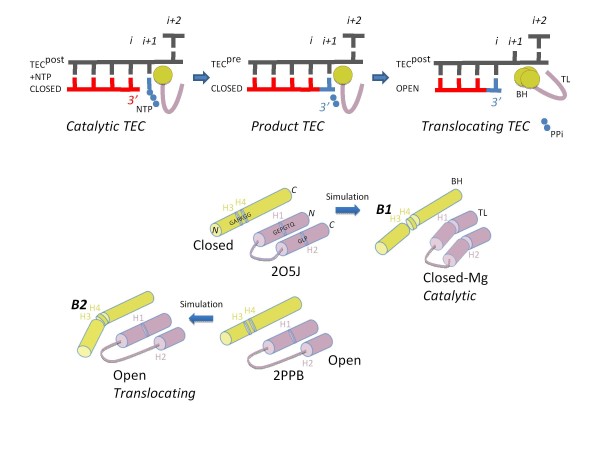
**A model for trigger loop hinges, bridge helix hinges and bridge helix bending modes based on all atom molecular dynamics simulations.** At the top of the figure, diagrams of the closed TEC, the closed product TEC (after chemistry) and the translocating TEC are shown. DNA is grey; RNA is red; the NTP substrate (or incorporated NMP and pyrophosphate) is blue; the trigger loop (TL) is purple; the bridge helix (BH) is yellow. Interpretations of simulations are shown schematically below. Simulations indicate trigger loop hinges H1 and H2, bridge helix hinges H3 and H4 and bridge helix bend modes B1 (straighter) and B2 (more sharply bent).

We compare short duration molecular dynamics simulations of Tt RNAP with closed and open trigger loop conformations to gain potential insight into mechanism and to generate predictions for site-directed mutagenesis. The RNAP TEC with a NTP bound and a closed trigger loop is thought to be the catalytic conformation, and the TEC with an open trigger loop is thought to approach the translocating form. Simulations have been run several times with consistent results, so representative trajectories are shown. A simulation of the closed catalytic TEC was done with restraints placed on the 3′-O of the RNA, the ATP substrate and Mg-I and Mg-II (referred to as “closed-Mg”). Restraining the active site to enforce catalytic geometry provides the greatest contrast to the open TEC simulation and, therefore, helps to generate useful predictions. Simulations indicate significant conformational flexibility in RNAP without large structural distortions. In particular, modeling indicates distinct bending modes in the H1-H2 trigger loop and H3-H4 bridge helix hinges, supporting our description of these motifs (Figure 1). Closed and open TEC simulations diverge in conformation and atomic contacts, suggesting targets for RNAP mutagenesis of hinges and of residues nearby and at a distance that may support hinge movement. We find that the simulations are highly predictive for the outcome of site-directed amino acid substitutions.

Although strongly predictive for mutagenesis, current models have limitations. The RNAP mechanism is complex involving phosphodiester bond synthesis (closed trigger loop), pyrophosphate release (closed→open trigger loop), translocation (open trigger loop), NTP-Mg loading (open→closed trigger loop). Because the bridge helix borders the active site, helix bending and dynamics are likely to influence multiple steps, including catalysis and translocation, and also to influence off pathway steps, such as stalling, pausing, arrest and termination. The current simulations relate most closely to two mechanistic steps, phosphodiester bond synthesis and translocation. Current models are not adequate in their design or duration to address the energetics or kinetics of transitions between steps. The entire RNAP bond addition cycle occurs on a time scale of ~1-50 milliseconds, so the current simulations (10.5 ns) provide limited snapshots of stages of the mechanism. The models give insight into partial trigger loop closing and opening, active site hydration/dehydration during catalysis and thermal ratchet translocation, which appears to be strongly restrained by tight trigger loop closing during catalysis and enabled by trigger loop opening and bridge helix bending at an N-terminal hinge(s) (H3/H4).

X-ray crystal structures of Tt RNAP TECs with a loaded ATP substrate and closed or open trigger loop conformations were analyzed by all atom molecular dynamics simulations in explicit solvent including water and counterions. For simulations, AMPcPP (α-, β-methylene ATP) in the original crystal structures was substituted with ATP. The extent of convergence of simulations from RMSD (root mean square deviation compared to the original crystal structure) is indicated in Additional file 1: Figure S1. Additional file 1: Figure S2 shows that simulations maintain reasonable geometries of nucleic acids and stable DNA/DNA and RNA/DNA base pairing. Most significantly, starting from very similar initial states, Tt RNAP TECs with closed or open trigger loops consistently diverged into distinct and recognizable conformations with characteristic alterations in functional atomic contacts.

Similarly to DNA polymerases (DNAPs), RNAPs utilize a two Mg^2+^ elongation mechanism [27,28]. In Tt RNAP, the metals at the active site are designated Mg-I and Mg-II, and in Sc RNAP II, the metals are designated Mg-A and Mg-B. It is likely that trigger loop closing, orientation of nucleotide substrates and associated metals, and dehydration of the active site are important aspects of DNAP and RNAP mechanism and fidelity [13,29]. A detailed model for the AMPcPP-Mg complex is presented in reference [16], which was used in our set up for a catalytic RNAP simulation with ATP substituted for AMPcPP. We find that binding Mg atoms to, 1) the RNA 3′-O, 2) ATP phosphate oxygens, and 3) key aspartic acid residues, results in a model that maintains catalytic geometry through simulation, as indicated by the RNA 3′-O to NTP α-phosphate distance and the separation of the active site Mg-I and Mg-II (Additional file 1: Figure S2B).

In this work, three representative RNAP simulations are compared: 1) a closed trigger loop TEC with bonded Mg atoms (with restraints) and loaded ATP (referred to as “closed-Mg”); 2) a closed trigger loop TEC lacking bonded Mg atoms (without restraints) and with loaded ATP (referred to as “closed”); and 3) an open trigger loop TEC without bonded Mg atoms (without restraints) and with loaded ATP (referred to as “open”). The comparisons, therefore, indicate the effects of restraining the catalytic center by bonding Mg atoms and also of trigger loop closing and opening. In the closed-Mg simulation, bonding of the Mg atoms was done to enforce a more precise catalytic geometry and also to maintain tighter trigger loop closing during simulation (Additional file 1: Figures S1 and S2). For balanced comparison, a closed TEC simulation is shown without bonded Mg atoms. The closed TEC simulation maintains a closed trigger loop conformation but diverges from catalytic geometry (Additional file 1: Figure S2). In Additional file 1: Figure S1, RMSD analyses indicate that, as expected, the closed-Mg TEC simulation remains most similar to the original 2O5J closed trigger loop TEC crystal structure. Additionally, bonding the Mg atoms restrains the motions of nucleic acids and ATP substrate (Additional file 1: Figure S1). Motions of the nucleic acids are most similar for the closed-Mg and closed RNAP TECs (Additional file 1: Figures S1 D-F), while overall motions of protein appear more similar for the closed and open trigger loop TECs (Additional file 1: Figures S1D-F), demonstrating that both restraining the active site Mg atoms and closing of the trigger loop are consequential in simulation.

A major conclusion of the molecular dynamics simulations is that the closed-Mg RNAP TEC shows significant conformational differences from the open TEC. Closed (2O5J) and open (2PPB) Tt RNAP TEC starting structures differ somewhat in the position of the AMPcPP substrate analogue (AMPcPP is found in insertion (closed; 2O5J) and pre-insertion (open; 2PPB) sites), the downstream DNA clamp, the “lid” and “rudder”. Overall, however, the original closed and open trigger loop TECs have a relatively straight conformation of the bridge helix and very similar conformations overall [16]. Notably, as simulations progress, much more dramatic differences develop than are present initially, comparing closed and open trigger loop RNAP TECs (Figure 1). Significant differences between these two conformations can be visualized readily in Movies M1 and M2. Supporting these distinct conformations, multiple ion pairs and hydrogen bonds in the two largest RNAP subunits switch atomic contacts in response to the conformation of the trigger loop. As expected, the closed TEC lacking bonded Mg atoms acquires an intermediate form that is generally most similar in conformation to the closed-Mg RNAP TEC.

At the top of Figure 1, three TEC intermediates are shown schematically: 1) a catalytic TEC with NTP substrate loaded; 2) a product TEC after phosphodiester bond formation but before trigger loop opening; and 3) a translocating TEC with an open trigger loop and bent bridge helix. The closed-Mg simulation appears to be a reasonable representation of the catalytic TEC. The closed TEC deviates from catalytic geometry during simulation and may represent a marginally opened intermediate in bond addition (i.e. before phosphodiester bond formation). The open TEC simulation is close to the translocating TEC except that our model includes an accurately loaded ATP substrate, which may restrict forward RNA/DNA translocation. After simulation, the closed-Mg TEC has a straight conformation of the bridge helix (B1 mode) with stretching between the H3 and H4 hinges, a tightly closed trigger loop and reasonable catalytic geometry (Additional file 1: Figure S2). The open TEC has a more bent conformation of the bridge helix (B2 mode), a relaxed and open trigger loop and a tendency for forward translocation (compare Movies M1 and M2).

### RNAP translocation

**Figure 2:**
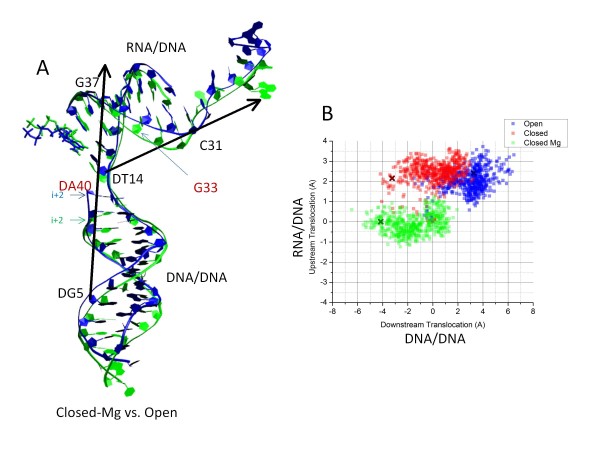
**The open trigger loop RNAP TEC appears to be the translocating conformation.** Vectors describe downstream DNA/DNA and upstream RNA/DNA translocation during simulation. The downstream translocation vector is drawn through DG5 (DNA template dGMP 5) and G37 (RNA GMP 37) 3′ carbons. Translocation is measured as the displacement of an orthogonal projection of DA40 (non-template DNA dAMP 40) 3′ carbon onto the vector relative to the initial structure. The upstream translocation vector is drawn through DT14 (DNA template dTMP 14) and C31 (RNA CMP 31) 3′ carbons. Translocation is measured as the displacement of an orthogonal projection of G33 (RNA GMP 33) 3′ carbon onto the vector relative to the initial structure. Closed-Mg (green) and open (blue) TECs are shown. B) Scatter plot of upstream and downstream translocation (each point represents a 20 ps interval). “X” indicates 10.5 ns time points for the three simulations relative to a (0,0) starting position in the original crystal structures (2O5J for closed; 2PPB for open).

Previously, normal mode analysis and molecular dynamics simulations of Sc RNAP II TECs with closed and open trigger loop conformations were compared to obtain insight into RNAP translocation [17,18]. Because the structure of a pre-translocated Tt RNAP TEC with an open trigger loop has not yet been reported, a post-translocated Tt RNAP TEC was selected with an open trigger loop and NTP in the active site (2PPB). The open RNAP TEC was considered to be a reasonable comparison to the closed-Mg and closed TEC simulations (2O5J), which also have a NTP loaded. In Figure 2, Additional file 1: S3 and S4, we compare the spontaneous translocation of nucleic acids during simulation of the closed-Mg, closed and open trigger loop Tt RNAP TECs. To determine the extent of nucleic acid displacement in each TEC, translocation vectors were defined for downstream DNA/DNA and upstream RNA/DNA (Figure 2A) [18]. Significantly, the downstream DNA/DNA is translocated forward in the open TEC compared to the closed-Mg TEC. As a dramatic example, the i + 2 downstream base-pair appears to be a full step forward (>3 Å) in the open TEC relative to the closed-Mg TEC. Notably, the upstream RNA/DNA region is relatively compressed in the closed-Mg TEC and more extended in the open TEC. In Additional file 1: Figure S3, relevant overlay snapshot comparisons of closed-Mg, closed and open TEC simulations are shown to indicate translocation dynamics during simulation.

In Figure 2B, a scatter plot is shown to compare translocation positions of closed-Mg, closed and open RNAP TECs. Consistent with snapshots (Figure 2A and Additional file 1: Figure S3), the closed-Mg TEC is the least forward translocated for downstream DNA/DNA (~ −2 Å on average) and upstream RNA/DNA (~ −0.5 Å) relative to initial crystal structures. Significantly, the open TEC is forward translocated for both downstream DNA/DNA (~ 3 Å) and upstream RNA/DNA (~ 2 Å). The closed TEC is intermediate between the closed-Mg TEC and the open TEC in downstream DNA/DNA translocation (~ 0 Å) and similar to the open TEC in upstream RNA/DNA expansion (~ 2.5 Å), emphasizing the role of the closed trigger loop in restricting translocation of the downstream DNA. Additional file 1: Figure S4 shows the trajectory of translocation vectors as a function of simulation time. Closing of the trigger loop has the effect during simulation of restricting the forward translocation of the downstream DNA/DNA duplex. Restraining the active site in the closed-Mg simulation has the effect of restricting the stretching of the upstream RNA/DNA hybrid.

The RMSD analysis shown in Additional file 1: Figure S1 also indicates that nucleic acids are much more mobile in the open trigger loop RNAP TEC than in closed TECs, consistent with the open trigger loop enabling forward translocation. These results are in excellent agreement with those of Feig and Burton for Sc RNAP II [17,18], although a closed-Mg TEC with active site restraints, which shows the greatest contrast from the open TEC in this work, was not simulated in the previous work. Taken together, our findings confirm the greater tendency for DNA/DNA and RNA/DNA forward translocation in the open TEC, which was predicted based on the previous molecular dynamics and crystal structure analyses [18]. In the open RNAP TEC, the RNA/DNA hybrid may be restrained from taking a full forward translocation step because of the presence of ATP, rather than incorporated 3′AMP and byproduct pyrophosphate, in the active site. In the open TEC simulation, the bridge helix sharply bends in a B2 conformation (Figure 1; Movie M2) and holds a B2 bend without oscillation. Simulation, therefore, may indicate a bridge helix bend associated with at least partial forward translocation. A similar conformational change has been suggested to initiate forward translocation of bacteriophage T7 RNAP after pyrophosphate release [30].

### RNAP hinges

**Figure 3:**
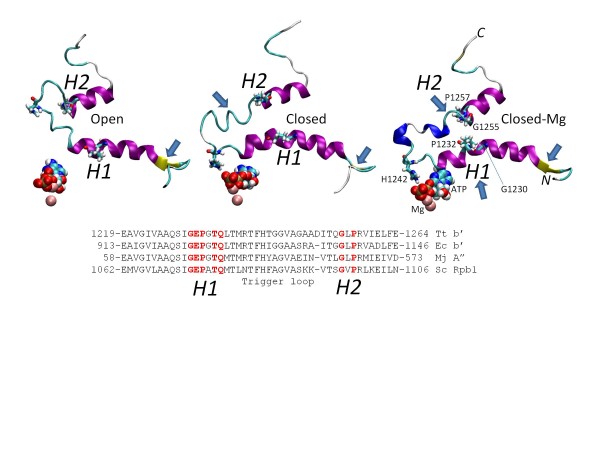
**The trigger loop hinges H1 (1230-GEPGTQ-1235) and H2 (1255-GLP-1257).** Snapshots of the trigger loop region in open, closed and closed-Mg TECs are compared at about 10 ns simulation. Coloring is in “secondary structure” representation: helix is magenta, 3_10_ helix is blue, coil is white, turn is cyan, β-sheet is yellow. G1230, P1232, H1242, G1255 and P1257 are in stick representation to locate the H1 and H2 hinges and orient the view. ATP and Mg are in space filling representation to locate the active site. Blue arrows indicate differences in secondary structure that develop during simulation.

Based on the flexibility of glycines and the helix breaking tendency of prolines, we propose that helices surrounding the flexible trigger loop include hinges H1 and H2 (Figures 1 and 3). H1 is predicted to lie within the sequence β’ 1230-GEPGTQ-1235, and H2 is predicted within the sequence β’ 1255-GLP-1257 [31]. The simulation reveals bending and hinge dynamics in the vicinity of G1230 (H1) and G1255 (H2). Movies M1 and M2 show trigger loop, bridge helix and nucleic acid dynamics for the closed-Mg and open RNAP TEC simulations. The movies provide a means to visualize motions of the H1 and H2 hinges. In Supplementary Materials, we show trajectory data (simulation time versus a dynamics metric (either ▵*psi* angle (a measure of amino acid residue conformation) or secondary structure)) indicating dynamic actions of H1 (Additional file 1: Figure S5) and H2 (Additional file 1: Figure S6). These graphs and the movies indicate that H1 and H2 are complex hinges with multiple modes of bending and dynamics depending on the state of the RNAP TEC and its position in the bond addition cycle.

**Figure 4:**
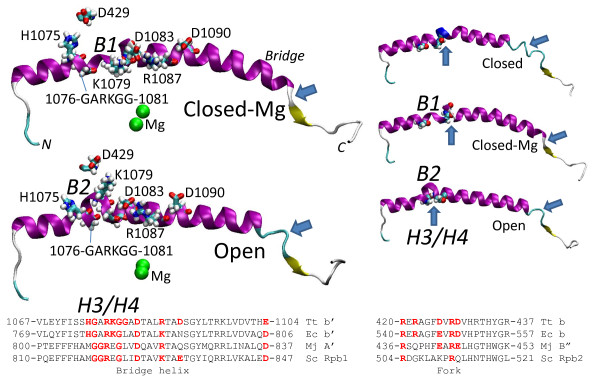
**The bridge α-helix hinges and bending.** To the right, 10 ns snapshots of closed, closed-Mg and open bridge helices are compared, colored in secondary structure representation, as in Figure 3. G1076, G1080 and G1081 are in space filling representation so that H3 and H4 hinges can be identified. To the left, proposed switch residues β’ H1075, K1079, D1083, R1087, D1090 and β D429 are shown in stick representation for closed-Mg and open TEC bridge helix structures. Blue arrows indicate differences in secondary structure that develop during simulations.

The trigger loop is in close association with the bridge helix [9]. Molecular dynamics simulations indicate that Tt RNAP β’ 1076-GARKGG-1081 on the bridge helix contains two nearby and semi-independent hinges described here as H3 (1076-GA-1077) and H4 (1080-GG-1081) (Figure 1). Flexibility in and around H3 and H4 glycines results in distinct modes of bridge helix bending referred to as B1 (closed-Mg TEC; a relatively straight bridge helix conformation) and B2 (open TEC; a strongly bent conformation) (Figures 1 and 4). The strongly bent B2 mode in the open TEC in particular might be expected to promote forward translocation of RNAP, consistent with an open TEC being a translocating conformation [17,18] (Figure 2, Additional file 1: S3 and S4). Distinct bridge helix bending modes B1 and B2 develop early in simulation and are stably maintained (Additional file 2: Movies M1 and Additional file 3: M2). In Additional file 1: Figure S7, trajectories of Δ*psi* versus simulation time and of secondary structure versus simulation time are shown for bridge helix hinges H3/H4. Additional metrics that indicate distinct bending and dynamics of trigger loop hinge H1 and bridge helix hinges H3/H4 comparing closed and open RNAP TECs are shown in Additional file 1: Figure S8. Additional file 1: Figure S8A indicates different bending modes of the H1 hinge comparing the closed-Mg to the closed and open TECs. Additional file 1: Figure S8B indicates different bending modes of the H3/H4 bridge helix hinges in the three simulations. We propose that motions and bending of hinges H1 to H4 initiates a larger conformational switch between catalytic (closed-Mg) and translocating (open) forms during each RNAP phosphodiester bond addition and nucleic acid translocation cycle. Slight bending of H1 and H2 coincides with acquisition of the catalytic conformation by the closed-Mg TEC. Mobility of the H2 hinge is pronounced both in the catalytic and translocating conformations, but may be specifically important for trigger loop opening (note the loss of the helical structure starting from H2 in the open versus closed conformations in Figure 3) [16,21].

### RNAP hinges in transcription

**Figure 5:**
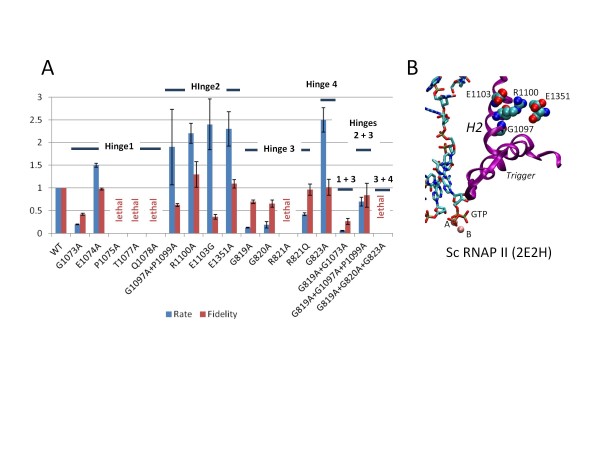
**Transcriptional activities of hinge H1 to H4 amino acid substitutions.** A) Elongation and fidelity assays. Blue bars represent transcriptional elongation rate relative to wild type RNAP II at 200 μM NTP concentration. Error bars indicate standard error from exponential curve fitting. Red bars represent transcriptional fidelity, determined by a competition assay (Additional file 1: Figures S9 and S10). Error bars are standard deviation of at least three independent determinations. B) Conserved ionic interactions surrounding the Sc RNAP II H2 hinge.

Because molecular dynamics simulation predicted that H1 to H4 are dynamic hinges, conserved hinge residues in *Saccharomyces cerevisiae* (Sc) RNAP II were substituted by in vitro mutagenesis and assayed for transcription rate and fidelity (Figure 5A). Mutations were made in Sc RNAP II rather than Tt RNAP for both pragmatic and tactical reasons. From a practical standpoint, we were initially better equipped to make the substitutions in Sc RNAP II. Furthermore, testing residues that are conserved across species provides more general insight into RNAP catalytic and translocation functions. Transcription rates are compared to wild type Sc RNAP II based on quantification of bulk elongation assays. The transcriptional fidelity analysis is based on a competition assay, in which AMP from ATP (the incorrect substrate at a high concentration) is misincorporated for GMP from GTP (the accurately templated substrate at a limiting concentration) (Additional file 1: Figures S9 and S10)) [32].

Mutations in the trigger loop hinge H1 (Sc RNAP II Rpb1 1073-GEPGTQ-1078) were expected to mainly affect formation of the catalytic conformation of the TEC because of its close proximity to the substrate NTP binding site and distinct conformational changes of the closed-Mg TEC observed in molecular dynamics simulations. Indeed, the G1073A substitution is very slow in elongation and demonstrates a significant fidelity defect (Figure 5A). Remarkably, P1075A, T1077A and Q1078A [2,33,34] are lethal substitutions in yeast, indicating essential functions. In Tt RNAP, Q1235A (corresponding to Sc RNAP II Q1078) is very slow in elongation and shows a strong fidelity defect [29]. G1076 substitutions were not made in this study because all possible homologous substitutions were previously reported for Mj RNAP and were shown to have a moderate effect on transcription [15]. Interestingly, E1074A appears faster than wild type Sc RNAP II in elongation, suggesting that E1074 might play a non-essential regulatory role that appears dispensable for maintenance of the catalytic conformation. The suggested regulatory role of E1074 does not appear to be related to mechanisms of cognate NTP selection, because this mutation does not affect transcriptional fidelity. Taken together, the results of H1 mutagenesis confirm the important role of the H1 hinge region in phosphodiester bond formation.

As mentioned above, hinge H2 appears to be important for trigger loop opening. Notably, delayed trigger loop opening has been proposed to impair NTP selection, decreasing transcriptional fidelity [2,10,20,34]. In agreement with the previously reported structural and functional evidence, substitutions in and around trigger loop hinge H2 of Sc RNAP II tend to be rapid in elongation and some show significant transcriptional fidelity defects. Sc RNAP II has the sequence 1097-GVPRLKE-1103, with major H2 bending predicted near to G1097. Significantly, substituting 1097-GVP-1099 to 1097-AVA-1099 generates a Sc RNAP II that is rapid in elongation with a moderate fidelity defect (Figure 5A). As previously reported and as confirmed here, E1103G, located near H2 is strongly defective in transcriptional fidelity [2,10,20]. E1103 forms an ionic contact to R1100, which forms an ionic contact to E1351 (Figure 5B). Similarly to the 1097-AVA-1099 H2 double substitution, R1100A, E1103G and E1351A appear to be rapid in elongation. R1100A and E1351A substitutions, however, do not render RNAP II error-prone, suggesting that transcription fidelity regulation may be restricted within the H2 hinge region, which includes G1097, P1099, R1100 and E1103. The gain of function (fast) phenotype of all the substitutions in and around H2 tested here suggests that transcription elongation rate may be regulated not only by H2 but also perhaps by downstream DNA contacts, which may be affected by H2 mobility. Because both G1097 and P1099 can be replaced with alanine, the mobility and bending of the H2 hinge observed in molecular dynamics simulations does not appear essential for formation of a catalytic RNAP conformation but rather may regulate the rate of phosphodiester bond formation and appears to play an important role in transcriptional fidelity.

All atom molecular dynamics simulations of Tt RNAP suggest that bridge helix hinges H3 and H4 (Sc RNAP II Rpb1 819-GGREGL-824) undergo extensive conformational changes in transition from catalysis to translocation. Sc RNAP II Rpb1 G819A and G820A are slow in elongation with moderate fidelity defects (Figure 5A), indicating that mobility of H3 contributes to an appropriate catalytic conformation. R821 is mounted between the clustered H3 and H4 hinges and connects the bridge helix with the Rpb2 fork region. R821A is a lethal substitution. R821Q is viable but slow in elongation. The phenotypes of R821 alleles suggest the functional importance of bridge helix interactions with the RNAP “fork” region (Tt RNAP β 420 to 450), which approaches the active site. G823A appears faster than wild type Sc RNAP II in elongation. The 819-GGREGL-824 → 819-AAREAL-824 triple alanine for glycine substitution in H3/H4 is a lethal substitution, suggesting the importance of flexibility and of the ability to deviate from precise α-helical structure in the H3/H4 hinge region. Extensive mutagenesis of the bridge helix H3/H4 region has been reported for Mj RNAP [14] and alanine scanning has been done for *Escherichia coli* (Ec) RNAP [35]. These previous studies, which also reported both fast and slow elongation phenotypes rendered by mutations in bridge helix residues are very consistent with the results reported here for Sc RNAP II H3/H4.

To test for possible synergy between RNAP trigger loop and bridge helix hinges, two additional substitutions were made. The Rpb1 G819A, G1073A double substitution combines a mutation in H3 (bridge) with one in H1 (trigger loop). In transcriptional activities, the effects of combining these two substitutions appear to be additive rather than synergistic. Similarly, the effects of combining G819A, G1097A, and P1099A substitutions in H3 (bridge) and H2 (trigger loop) also appear to be approximately additive in transcriptional effects. These results are most consistent with hinges on the trigger loop and the bridge helix being largely independent of one another in the RNAP bond addition cycle, because tight coupling of trigger loop and bridge helix hinge motions might be expected to produce synergistic effects of combining hinge mutations. In the work of others, multiple RNAP mutations have been combined around the bridge helix C-terminal glycine hinge of Mj RNAP (G825) [15] and within the trigger loop region of Sc RNAP II [2].

### Exonuclease III mapping

**Figure 6:**
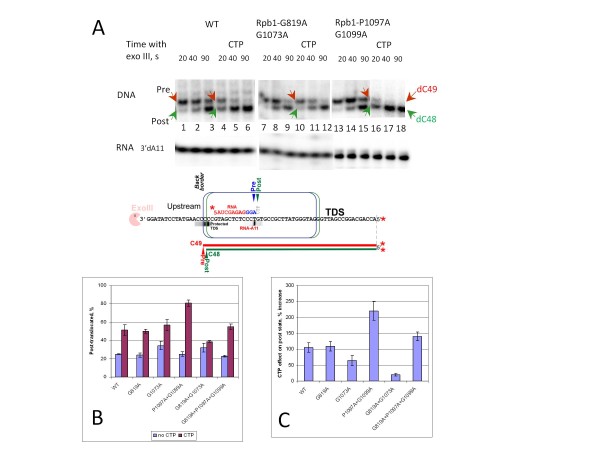
**Exonuclease III mapping of Sc RNAP II TECs.** A) Back border (upstream border) 3′-dA11 Sc RNAP II TEC mapping. 3′-dA11 (3′-dATP chain terminated A11 TECs) RNA controls, run in the same reaction, are shown to demonstrate effective chain termination at the A11 position. The strategy for mapping Sc RNAP II TECs in the absence or presence of incoming CTP is indicated at the bottom of the figure. B) Phosphorimager quantification of triplicate determinations done at 30 s exonuclease III incubation. C) Percent increase in the post-translocated state induced with CTP compared to wild type Sc RNAP II, from the data shown in B. Error bars represent standard deviation.

To further investigate possible interactions between RNAP hinges, exonuclease III mapping was done from the upstream RNAP border for a 3′ chain-terminated A11 TEC in the absence and presence of the incoming NTP (Figure 6). A schematic representation of the 3′→5′ exonuclease III back border mapping experiment is shown at the bottom of Figure 6A. Mapping provides insight into the resting translocation states of the Sc RNAP II TEC and the effects of the incoming cognate NTP on translocation [20,36]. There is a strong correlation between the stable sequestration of the cognate NTP in the RNAP II active site and the magnitude of the incoming NTP effect on the pre- to post-translocated border transition in the exonuclease III assay [20,36], showing that the post-translocated state is stabilized by binding of the cognate NTP in the active site [8]. Alternatively, the apparent stimulation of translocation might reflect an allosteric effect of the incoming NTP previously proposed for mammalian RNAP II [4,37] and Ec RNAP [6,7,38].

In the experiment shown in Figure 6, the cognate CTP (encoded at C12) promotes the pre-to-post-translocated border transition in 3′dA11 chain terminated Sc RNAP II TECs (Figure 6A, compare lanes 1–3 and 4–6 and Figures 6B-C; note that in Figure 6C the approximately 2-fold increase of the post-translocated state for wild type RNAP II corresponds to the increase of the post-translocated fraction from 25% to 51% as shown in Figure 6B). G819A substitution targeting hinge H3 does not affect RNAP II interaction with the incoming CTP (Figures 6B-C). The G1073A substitution in the trigger loop hinge H1 leads to an apparent decrease of the incoming CTP effect on translocation. This effect may be due to the post-translocated shift of 3′dA11 TEC equilibrium as compared to the wild type TEC (Figure 6B, compare the wild type and G1073A RNAP II without CTP), but not to a defective interaction of G1073A RNAP II with the incoming CTP (Figure 6B, compare the wild type and G1073A in the presence of CTP). Notably, the combination of mutations in the trigger loop hinge H1 and bridge helix hinge H3 practically abolishes the ability of the incoming CTP to stabilize the post-translocated state (Figure 6A, compare lanes 4–6 and 10–12; also Figure 6B-C). Because these two mutations have apparently additive effects on the elongation rate and fidelity of RNAP II (Figure 5A), their combined effects on NTP stabilization of the post-translocated TEC (Figure 6) may reflect the complexity of the RNAP elongation cycle or allosteric effects of NTPs. The translocation assay in the presence of the cognate NTP indicates a synergistic action of H1 and H3 hinges in NTP binding. Unlike H1, the distal trigger loop hinge H2 does not appear to have synergy with bridge helix hinge H3 in the exonuclease III-based translocation assay. In agreement with the results of the elongation and fidelity assays, which demonstrate a gain of function phenotype in elongation and decreased transcriptional fidelity, mutations in trigger loop hinge H2 (G1097A, P1099A) increase the effects of the incoming CTP on the translocation equilibrium, further supporting the importance of H2 in cognate NTP selection by RNAP [20]. The significant effect of the bridge helix hinge H3 G819A substitution on translocation in the presence of either a trigger loop hinge H1 G1073A single or a H2 1097-GVP-1099 → 1097-AVA-1099 double substitution (Figure 6B-C) indicates that the bridge helix H3 hinge region may have a role in translocation, as indicated in simulations (Movies M1 and M2).

Importantly, the effects of mutations in the hinges on translocation have been pronounced only in the translocation assays performed in the presence of the incoming NTP. This result may indicate that mutations in the hinges largely affect trigger loop and bridge helix interactions with the NTP positioned for catalysis in the active site of the closed TEC. Alternatively, it cannot be excluded that translocation of the stalled TEC deprived of NTPs and translocation during ongoing transcription, which takes place in the presence of the incoming NTP, occur by distinct pathways. Notably, very recent optical trapping single molecule experiments suggest that the NTP can bind both the pre- and post-translocated TECs [10].

### Hinge conformations appear to be supported by alternate switch residue contacts

Different modes of hinge H1 to H4 action and B1 and B2 bridge helix bending result in long-range changes in the atomic contacts of amino acid residues of Tt RNAP β and β’ subunits. We refer to the RNAP amino acids making altered contacts as “switch” residues. Note that these residues should not be confused with disordered segments of RNAP chains that assume increased structural order upon nucleic acid binding (those segments of chain previously designated “switches 1-5”) [12,39]. In this paper, switch residues are amino acid side chains predicted from molecular dynamics simulation to alter their molecular contacts (ionic contacts or hydrogen bonds) in response to closing and opening of the trigger loop.

Molecular dynamics simulation reveals a charge relay chain across the bridge helix involving Tt RNAP β’ H1075, K1079, D1083, R1087, D1090 and fork residue β D429 (Figure 4, left images). Additional file 1: Figures S11-S13 show atomic contacts and simulation trajectories that relate to the proposed switching mechanism. β’ K1079, which is mounted between the H3 and H4 hinges, and β D429, with which K1079 can interact (open TEC), appear to be central residues in supporting different conformations of the bridge helix and interacting fork. Comparing late simulation snapshots of closed-Mg and open TECs, K1079 switches its contacts between D1083 (closed-Mg) and D429 (open TEC). Tt RNAP K1079 and D429 are not conserved in Sc RNAP II, indicating that the core of this switching mechanism is specific to bacterial RNAPs. Tt RNAP β’ D1083, R1087 and D1090 in the charge relay chain, however, are conserved in evolution and are expected to fulfill a similar role in both Tt RNAP and Sc RNAP II. The homologous substitution to Tt RNAP β’ K1079A has been made in Ec RNAP and is strongly defective in elongation [35], as we would predict based on the central role of this switch residue in simulations. R1078, adjacent to K1079 and mounted between the bridge helix H3/H4 hinges, is also important in regulating the structure of the fork (Additional file 1: Figures S11-S13). Interestingly, Tt β’ D1090 is substituted with asparagine in Ec RNAP and glutamine in Mj RNAP, changes that would diminish interactions with R1087. Notably, Mj RNAP Q→E/D substitutions at the position corresponding to Tt RNAP β’ D1090 are strong gain of function mutants (rapid in elongation) [14], consistent with the predicted importance of Tt β’ D1090 in supporting bridge helix bending and dynamics.

**Figure 7:**
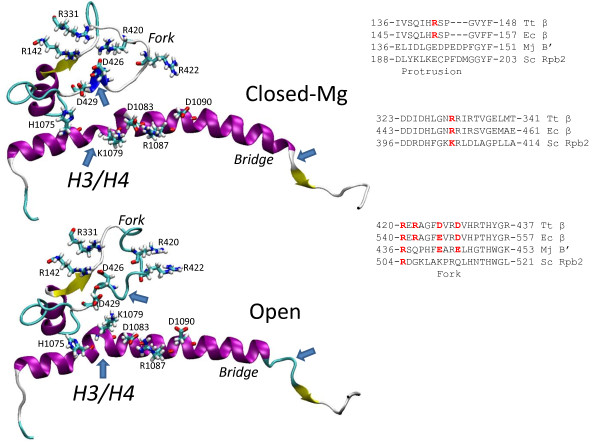
**Proposed interactions between bridge helix switch residues β’ H1075 and K1079 and the fork region.** Fork residues β R420, D426 and D429 are proposed from simulations to be switch residues. Interacting residues β R142 and R331 also appear to be involved in the switching mechanism.

Based on all atom simulations, predictions for the Tt RNAP fork region and its contacts can potentially be extended. Specifically, Tt RNAP β R142, R331, R420, D426 and D429 are predicted to make potentially significant switching contacts (Figure 7). R142, R331 and potentially R420 can interact with D426, which is close to D429, which in the open TEC appears to make a key interaction to β’ K1079 on the bridge helix (Figures 7 and Additional file 1: Figures S11-S12). We posit that maintaining different ionic contacts to D426 by R142, R331 and R420 reorients D429 to approach bridge helix residue H1075 (closed-Mg) or K1079 (open). If H1075 can take on a +1 charge by protonation, H1075 could form an ion pair with D429 in the closed-Mg TEC.

### A conserved switch near the RNAP active site

**Figure 8:**
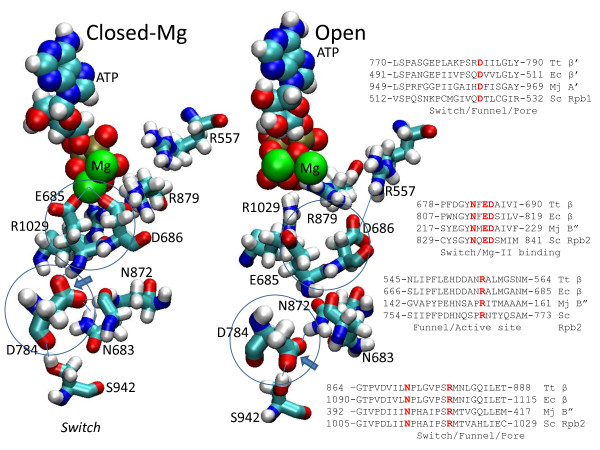
**A conserved conformational switching mechanism centered on β’ D784 about 20 Å from H1.** The substrate ATP, Mg-I and Mg-II (green) are in space-filling representation. Proposed switch residues are in stick representation. The two orientations of D784 observed in simulations of closed and open trigger loop RNAP TECs are indicated with blue arrows. Some atomic interactions are indicated with blue lines or emphasized with blue circles.

From simulations of open RNAP TECs, a conserved switch is predicted beneath the Tt RNAP active site near Mg-II centered about 20 Å from the H1 trigger loop hinge (Figure 8). Additional file 1: Figures S14-S17 show atomic contacts that may be altered comparing the closed-Mg and open TEC RNAP snapshots. Tt RNAP β E685 and D686 appear invariant in evolution and closely approach Mg-II in x-ray crystal structures [40-42]. The central residue in the predicted switch is β’ D784. We posit that a potential role of this switch may be in release of the RNAP reaction byproduct pyrophosphate (diphosphate) and Mg-II after phosphodiester bond formation. Most significantly, in all closed RNAP TEC simulations, D784 maintained a distinctly different set of molecular contacts (more similar to the initial x-ray crystal structure) than those that develop during simulation of the open TEC. The images in Figure 8 are representative of multiple simulations. Remarkably, the carboxyl head group of β’ D784 points up (in this view) toward the main chain NH of β E685 to form a hydrogen bond in the closed-Mg RNAP TEC. In the open TEC, by contrast, the carboxyl head group of β’ D784 is consistently switched downward toward the β’ S942 hydroxyl head group to form a hydrogen bond. These interactions involve contacts of β’ D784 with β N683 and β N872. Only in the open TEC, an ion pair develops between β D686 and β R557, after D686 and E685 dissociate from Mg-II (Figures 8 and Additional file 1: S14-S17). These simulations, therefore, predict that β’ D784 and S942 and β R557, N683, E685, D686 and N872 are switch residues participating in interactions with Mg-II that alter atomic contacts between the catalytic and translocating conformations of the Tt RNAP TEC; β’ D784 appears to be of central importance in operating the switch.

**Figure 9:**
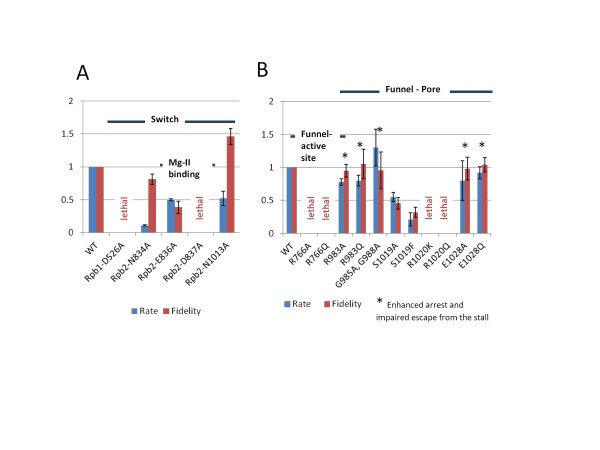
**Transcriptional activities and fidelity of Sc RNAP II substitutions in the active site and proposed switch residues (as in Figure**5A).

Site-directed mutagenesis of homologous switch residues identified in simulations and a number of nearby active site residues was done in Sc RNAP II (Figure 9). Consistent with the proposed central role for D784 in this switch, Sc RNAP II Rpb1 D526A, corresponding to Tt RNAP β’ D784, is a lethal substitution. Sc RNAP II Rpb2 N834A, corresponding to Tt RNAP β N683, is very slow in elongation, consistent with its proposed role supporting the switch. Sc RNAP II Rpb2 N1013A, corresponding to Tt RNAP switch residue β N872, is slow in elongation and appears to have enhanced transcriptional fidelity relative to wild type Sc RNAP II.

Consistent with the results of others, Sc RNAP II Mg-B interacting residues Rpb2 E836 and D837 (corresponding to Tt RNAP β E685 and D686) substitutions have significant effects on transcription [40-42]. Rpb2 D837A is a lethal substitution. Rpb2 E836A is slow in elongation and defective in transcriptional fidelity. The defect in E836A fidelity can be attributed to misalignment of Mg-B and, thus, the substrate NTP during phosphodiester bond formation. Other substitutions that are expected to misalign the NTP substrate are Rpb2 S1019A and S1019F, which are adjacent to R1020, which corresponds to Tt RNAP β R879 (Figure 8). Sc RNAP II Rpb2 R1020K/Q substitutions are lethal (Figure 9) [43], indicating an essential function. The bulkier S1019F substitution is more strongly defective in transcription and fidelity than the S1019A substitution, as expected, if adjacent R1020 orients the NTP substrate for catalysis through its contact to the NTP γ-phosphate. Sc RNAP II Rpb2 R766A/Q substitutions, corresponding to Tt RNAP β R557, are lethal (Figure 9) consistent with simulations (Figures 8 and Additional file 1: S17) [25].

Other substitutions, addressing the functional roles of the Rpb2 subunit switch residues located in the funnel and pore regions of the Sc RNAP II TEC are described in more detail in reference [43]. R983 is expected to form an ionic contact with E1028, and G985 and G988 ensure the flexibility of R983. Note that enhanced propensity to undergo transcriptional stalling or arrest (i.e. by backtracking) observed in Rpb2 mutants R983A, G985A, G988A and E1028Q may indicate a defect in forward translocation of the stalled TEC. These mutations might also affect RNAP interactions with the 3′ RNA extruded from the active site during backtracking, stabilizing the arrested conformation of the TEC.

### Active site dehydration in catalysis

In x-ray crystal structures, often water that is present within a protein is not resolved. Molecular dynamics simulation in explicit solvent, however, generates a model for internal hydration of a protein. Previously, trigger loop closing was posited to drive water from the RNAP active site and to order water to optimize conditions for catalysis [13]. This model for active site closing and dehydration to order water is potentially a powerful and general concept to describe catalysis within enclosed active sites of enzymes [44-47]. In support of such a model for RNAP active site dehydration during trigger loop closing, the closed-Mg, closed and open RNAP TECs are compared for active site hydration (Additional file 1: Figures S18-S20). At the ATP N3 position, the closed-Mg TEC remains strongly dehydrated throughout the simulation (Additional file 1: Figure S18A). The closed TEC, which deviates from catalytic geometry (Additional file 1: Figure S2) and may not be as tightly closed as the closed-Mg TEC, is slightly more hydrated. As expected, the open trigger loop, which admits more access to water, is much more strongly hydrated around ATP N3. Measuring water surrounding the RNA 3′-O, similar results are obtained (Additional file 1: Figure S18B). We conclude that at least one function of trigger loop closing is to reduce the hydration of the NTP substrate and to enhance the ordering of waters in the active site. Tightly bound waters are detected at Mg-I in both closed-Mg (one bound water) and open (three bound waters) RNAP TEC simulations (Additional file 1: Figures S19-S20).

### Model for RNAP cycling between catalysis and translocation

From all atom molecular dynamics simulations of Tt RNAP TECs, we posit that RNAP may cycle during elongation between at least two distinct conformations that support either catalysis or translocation. The two conformations are characterized by bending at hinges within helical segments surrounding the trigger loop (H1 and H2) and toward the N-terminal end of the bridge helix (H3/H4) (Figures 1,3 and 4). Hinge motions are supported by amino acid “switch” residues (distinct from RNAP “switch regions 1-5”) that change atomic contacts in response to hinge action. Remarkably, these switch residues can be located at significant distances from the hinges and from the RNAP active site. The catalytic TEC appears to resist forward translocation because of the tight closing of the trigger loop over the NTP substrate. By contrast, the RNAP TEC with an open trigger loop appears to support increased forward nucleic acid translocation and enhanced nucleic acid motion. Furthermore, in the RNAP TEC with an open trigger loop conformation, the bridge helix becomes more strongly bent against the RNA/DNA hybrid in simulation, indicating that mechanical pushing by the bridge helix may contribute to a fractional forward translocation step. In current simulations performed on a nanosecond time-scale, there is little indication of an oscillating RNAP ratchet that might drive both forward and reverse translocation and unrestrained nucleic acid sliding. Rather, bridge helix bending against the RNA/DNA hybrid appears to generate a more deliberate but partial forward step. Very similarly, during movement of bacteriophage T7 RNAP, a steric clash develops between the pre-translocated RNA/DNA hybrid and the open product RNAP TEC stimulating forward translocation [30].

Based on mutagenesis of conserved residues in Sc RNAP II, we propose that important aspects of this hinging and switching mechanism are strongly conserved in evolution. Tt RNAP appears to have two specialized hinges H1 and H2 within helical segments surrounding the mobile trigger loop that are strongly conserved in other RNAPs. Mutation of the H1 and H2 trigger loop hinges in Sc RNAP II strongly supports their importance in transcription. Most H1 substitutions are loss of function mutations, but substitutions in and around H2 tend to be gain of function mutations that can be rapid in elongation and also error-prone (Figure 5). Hinges H3 and H4 clustered toward the N-terminal end of the bridge helix appear to induce bending modes B1 (straighter) and B2 (more strongly bent) to support catalysis and translocation. Essentially all RNAPs appear to have a flexible H3/H4 bridge helix hinge region, but H3/H4 glycines may be differently distributed in different RNAPs, indicating that somewhat different bends, flexibility and dynamics may be emphasized for different RNAPs. Because bridge helix flexibility and bending appears to contribute to multiple steps of the bond addition cycle, indicating multiple bending modes, full conservation of H3/H4 across RNAPs of different classes and species is not expected. The wide distribution of apparent switch residues in Tt RNAP appear to indicate a long range conformational change associated with trigger loop closing and opening and progression through each bond addition, pyrophosphate release and translocation cycle.

Mutagenesis of a conserved switch that may have a role in pyrophosphate release, located about 20 Å from the trigger loop H1 hinge, supports the importance of switch residues in transcription elongation. Some other switch residues that are predicted from simulations are specific to bacterial RNAPs, and those that have been altered appear important for transcriptional functions. In some cases, the severity of RNAP mutations appears to reflect the roles of residues predicted from our simulations. As an example, Ec RNAP β’ K781A, corresponding to key switch residue Tt β’ K1079 (Figure 4), is strongly defective for transcription compared to surrounding substitutions (Table 1) [35]. For multi-subunit RNAPs, therefore, molecular dynamics simulation can be used to predict conformational changes in the RNAP TEC and the potential functions of key amino acid residues.

**Table 1:** **Summary of mutations. 6-AU**^**s **^**indicates sensitivity to 6-azauracil**

**Structural location**	**Subunit, residue and mutation**			**Activity in Sc (Tt or Ec)**		**References**
	**Tt RNAP**	**Ec RNAP**	**Sc RNAP II**	**in vivo**	**in vitro**	
Switch residue	β R557	β R678C	Rpb2 R766Q/A	Lethal	(Ec minor defects)	[13,38]
Switch residue	β N683	β N811	Rpb2 N834A	6-AU^s^	Very slow, moderate fidelity defect	this paper
Mg^2+^ binding	β E685	β E813	Rpb2 E836A	6-AU^s^	Slow, severe fidelity defect (Ec slow elongation)	this paper [40,42,48,49]
Mg^2+^ binding	β D686	β D814	Rpb2 D837A	Lethal	(Ec slow elongation)	this paper [42,48]
Switch residue	β N872	β N1099	Rpb2 1013A		Slow	this paper
γ-phosphate	β S878	β S1105	Rpb2 S1019F	6-AU^s^	Slow, severe fidelity defect	this paper
γ-phosphate	β R879	β R1106	Rpb2 R1020K/Q	Lethal		[43]
Switch residue	β’ D784	β’ D505	Rpb1 D526A	Lethal		this paper
H3/H4 hinges, bridge helix			Rpb1 G819A, G820A, G823A	Lethal		this paper
H3 hinge, bridge helix	β’ G1076	β’ G778	Rpb1 G819A		Slow, minor fidelity defect	this paper, [14,35]
H3 hinge, bridge helix	β’ A1077	β’ A779	Rpb1 G820A		Slow, moderate fidelity defect	this paper, [14,35]
H3/H4 hinges, bridge helix	β’ R1078	β’ R780C	Rpb1 R821Q/A		R821Q slow, R821A lethal (Ec termination defects)	this paper, [50][14,35]
H3/H4 hinges, bridge helix	β’ K1079	β’ K781A	Rpb1 E822		(Ec very slow elongation)	[35]
H4 hinge, bridge helix	β’ G1080	β’ G782	Rpb1 G823A		Little effect, moderate fidelity defect	this paper, [14,35]
H1 hinge, trigger loop			Rpb1 G1073A, P1075A	Lethal		this paper
H1 hinge, trigger loop	β’ G1230	β’ G924	Rpb1 G1073A		Slow, strong fidelity defect	this paper, [2]
H1 hinge, trigger loop	β’ E1231	β’ E925	Rpb1 E1074A		Fast	this paper, [2]
H1 hinge, trigger loop	β’ P1232	β’ P926	Rpb1 P1075A	Lethal		this paper, [2]
H1 hinge, trigger loop	β’ T1234	β’ T928	Rpb1 T1077A	Lethal		this paper, [2]
H1 hinge, trigger loop	β’ Q1235A	β’ Q929	Rpb1 Q1078A	Lethal	(Slow, error-prone in Tt)	this paper, [2,29,33]
H2 hinge, trigger loop			Rpb1 G1097A, P1099A		Rapid in elongation, moderate fidelity defect	this paper, [2]

In simulation of the closed-Mg Tt RNAP TEC, we constructed extra bonds between the Mg atoms, NTP phosphate oxygens, the 3′-O of the RNA chain and catalytic aspartic acid residues. These modifications appeared to tighten trigger loop closing and to support reasonable catalytic geometry (Additional file 1: Figures S1-S2, Movies M1-M2). Another group sought in simulations to tighten Sc RNAP II trigger loop closing by protonation of a trigger loop histidine (Sc RNAP II Rpb1 H1085; Tt RNAP β’ H1242) [25,26], but we did not adopt this strategy because we were unsure how to predict the appropriate protonation state of this histidine in closed and open RNAP TECs. During simulation, bonding Mg atoms and enhancing trigger loop closing resulted in strongly restrained forward translocation and excluded water from the RNAP active site (Additional file 1: Figures S18-S20). It appears that our closed-Mg simulation provides a reasonable initial model for a catalytic Tt RNAP TEC and an appropriate comparison to closed and open RNAP TEC simulations.

The simulation of an open trigger loop TEC provides a comparison to the closed-Mg model but was not set up as a perfect representation of a translocating TEC. For one thing, an ATP substrate is accurately loaded in the active site, which may restrain forward translocation. Normally, a translocating RNAP TEC would be a product complex with an open trigger loop, an incorporated NMP and a released pyrophosphate (see Figure 1). Comparing the simulations, the open trigger loop TEC has more relaxed trigger loop hinges H1 and H2 compared to the closed-Mg TEC, for which much more action of the H1 and H2 hinges is evident (compare Movies M1 and M2). Interestingly, a sharp bridge helix H3/H4 B2 bend develops very early during simulation of the open trigger loop RNAP TEC and is stably maintained without observed B2←→B1 or B2←→initial conformation (2O5I or 2PPB) oscillations. The B2 bending mode is expected to generate mechanical translocation force on the RNA/DNA hybrid, which appears to be indicated by strong forward thrust of the DNA/DNA duplex and expansion of the RNA/DNA hybrid during open TEC simulation (Figures 2 and Additional file 1: S3-S4). Based on the enhanced bending of the bridge helix (B2 mode) observed for the Tt RNAP open trigger loop TEC compared to a straighter bridge helix in the closed TEC (B1 mode) (Figure 4, Additional file 2: Movies M1 and Additional file 3: M2), we support the proposal previously made by others that bridge helix bending against the RNA/DNA hybrid may help to stimulate forward translocation [12]. Because the translocation step is short (about 3.4 Å), limited motions of the bridge helix followed by nucleic acid sliding may largely account for the precise forward step of RNAP. The proposed translocation mechanism should limit RNAP to single base steps because the mechanical contribution to the step appears to be fractional, making hyper-translocation unlikely. Nucleic acid sliding in the presence or absence of the incoming NTP could account for the remainder of the forward step. If the incoming NTP is present, the trigger loop is expected to close, preventing hyper-translocation.

Based on simulations, it appears that a set of hinges H1 to H4 within helical segments surrounding the trigger loop (H1 and H2) and proximal to the N-terminal end of the bridge helix (H3/H4) are important in catalysis and translocation. Somewhat surprisingly, combining mutations in multiple RNAP hinges appears to give additive rather than synergistic transcriptional effects (Figure 5), although exonuclease III mapping may indicate somewhat greater functional coupling of hinges, at least in the presence of an incoming NTP (Figure 6). So far, functional assays appear to indicate that hinges H1-H4 function somewhat independently during the elongation bond addition cycle. Hinges H3 and H4 are very close together on the bridge helix, and it is not yet clear whether H3/H4 should be considered to be one complex hinge or two semi-independent hinges. In the future, other bending modes of the bridge helix may be detected as new simulations and x-ray crystal structures become available.

## Conclusions

Using all atom molecular dynamics simulations, comparison of Tt RNAP TEC structures was used to generate a model for cycling between catalytic and translocating conformations during elongation. The catalytic TEC appears to have a tightly closed trigger loop and, as a result of loop closure, a relatively dehydrated active site. Tight trigger loop closure appears to be facilitated by GXP hinges within α-helices at the base of the loop. Near the N-terminal end of the bridge α-helix, a complex glycine hinge(s) also appears to support the catalytic RNAP structure by maintaining a relatively straight but somewhat extended bridge helix conformation.

By contrast, the open conformation of the trigger loop supports translocation. In the open conformation, GXP trigger loop hinges are relatively relaxed, and the more C-terminal GXP hinge (H2) becomes the site of helix breaking, increasing the size of the loop. During simulation of the open trigger loop RNAP TEC, the bridge helix N-terminal hinge(s) bends against the RNA/DNA hybrid stimulating forward translocation. In simulation, switching between catalytic and translocating RNAP TECs is supported by amino acid residues that trade atomic contacts. Mutation of hinge and switch amino acid residues and assay of mutant RNAPs supports the proposed model. A conserved switch is identified by simulation, mutation and assay that appears to function in pyrophosphate release after catalysis and trigger loop opening.

## Methods

### Tt RNAP TEC simulations

Molecular dynamics simulation of Tt RNAP closed trigger loop TECs (PDB 2O5 J with some missing chain taken from PDB 2O5I) in explicit solvent with counterions was done as previously described [13]. AMPcPP (α-, β-methylene ATP) in the original crystal structures was substituted with ATP for simulation. During modeling, Tt RNAP β’ H1242 was not protonated because we were not certain how to predict the protonation state of this residue [25,26]. Thio-substitutions in nucleic acids used to block RNA cleavage by Tt RNAP in crystals were not present in the protein data bank files and were not considered in modeling [16,21]. For the closed-Mg simulation, extra bonds were assigned according to reference [16]. For this simulation, Mg-I was covalently bonded to the RNA 3′O, β’ D739 OD1, β’ D741 OD1, β’ D743 OD2 and the α-phosphate O. Mg-II was covalently bonded to the α, β and γ-phosphate oxygens and to β’ D739 O2. Bond lengths were taken from the original 2O5J crystal structure and were maintained with a force constant of 200 kcal/mol/Å^2^. For the closed TEC simulation, extra bonds to Mg atoms were omitted. Simulation of the open trigger loop TEC (PDB 2PPB) was done by essentially the same protocol as the closed trigger loop TEC. For consistency, streptolydigin, which was present in 2PPB but absent from 2O5J, was omitted in simulation of the open TEC. Missing chain in the 2O5J closed trigger loop TEC cleft region was patched with chain from the 2O5I TEC structure using the program MODELLER [51]. Simulations were done on the NSF XSEDE (Extreme Science and Engineering Discovery Environment) national computer system using the University of Texas, Austin, Sun Constellation Linux Cluster TACC (Texas Advanced Computer Center) Ranger computer. To parallelize large computer jobs, simulations were done using the program NAMD (Not (just) Another Molecular Dynamics Program) [24] using AMBER (Assisted Model Building with Energy Refinement) [23] force fields. To limit computation time, α and ω subunits were omitted from the simulated files, as previously described [13]. Because no large distortions were observed near omitted α and ω subunits, these deletions in the TECs did not appear to negatively affect simulations. Potentially, however, conformational changes observed in simulations may be accelerated or amplified by omission of α and ω subunits. Additional simulations with all RNAP subunits have now been run but for simplicity of presentation are not reported here. These simulations produce closed-Mg, closed and open RNAP TEC conformations that are very consistent with those shown here.

Equilibration consists of 500–1000 steps of minimization followed by instantaneous heating to 320 K and a gradual release of harmonic restraints from 5 to zero kcal/mol/Å^2^ over 500 ns. This is followed by 10.5 ns of production simulation run at 2 fs/step with the SETTLE algorithm and TIP3P water. A Langevin thermostat and barostat are used to maintain 320 K temperature and 1 atm pressure. Simulations were run using NAMD version 2.8 and with Amber ff10 force fields from Amber 11. Non-bonded (electrostatic and Van der Waals) forces are calculated out to 10 Å with switching at 8 Å for smoothing the cutoff. For the calculation of electrostatic forces, Particle Mesh Ewald summation is used with periodic boundary conditions [23,24,52].

Molecular images were prepared using the program Visual Molecular Dynamics (VMD) [53]. Representative PDB files from simulations will be made available on request to ZFB (burton@cns.msu.edu).

### Strains and in vitro mutagenesis

Site-directed mutagenesis, production and purification of Sc RNAP II mutant proteins was done essentially as previously described for *rpb2* mutants [43]. For production of *rpb1* mutant strains a similar set of plasmids and strains was constructed. Phenotypic testing of yeast strains was as described [43].

### In vitro transcription

Bulk elongation assays were done as previously described [54]. Briefly, TECs were assembled by annealing - RNA oligonucleotide (5′ AUCGAGAGG 3′) to the template DNA oligonucleotide (5′ GGT TTG CCC CGT TGG ACG TGT GGA TTG GGA TGG CTA TTC GCC GTG TCC CTC TCG ATG GCT GTA AGT ATC CTA TAG G3′), and incubation of Sc RNAP II with the RNA-DNA hybrid followed by addition of the non-template DNA strand (5′ CCT ATA GGA TAC TTA CAG CCA TCG AGA GGG ACA CGG CGA ATA GCC ATC CCA ATC CAC ACG TCC AAC GGG GCA AAC CGT A 3′). The RNA in the resulting TEC was labeled by 2 min incubation with 0.15 μM α-^32^P GTP, and the resulting G10 TEC was purified from the unincorporated GTP and nucleic acids using the centrifugal filtration through a 100 kDa cutoff membrane. Transcription was done with 200 μM NTPs, and the time points were 5, 10, 20 and 40 s. For most mutants the experiment was done at least twice. Error bars represent standard error from a single phase exponential fit of the runoff accumulation time course.

### Fidelity assays

Competition fidelity assays were done as in Walmacq et al., 2012 [32] and as shown in Figures S9 and S10. Accurate GMP incorporation at G10 is discriminated from misincorporation of AMP at A*10 (* to indicate the misincorporation product) by the different mobilities of the short RNAs on gels. The results of this assay and the calculated fidelities are very similar to those obtained using more complex normalization protocols [20] (Additional file 1: Figure S10).

### Exonuclease III footprinting of RNAP translocation state

Sc RNAP II was isolated and assembled into G9 TECs, as described [20,43]. TECs were extended to G10 and A11 with addition of 10 μM GTP and 100 μM chain terminator 3′-dATP. Ec exonuclease III (New England Biolabs; 3.3 U/μl) mapping of the upstream Sc RNAP II TEC border was done as described in Kireeva et al. [20]. Exonuclease III digestions were done at 25 C. DNA and RNA were resolved in 10% polyacrylamide-7 M urea gels. Quantification of ^32^P in wet gels was done using a Typhoon 9200 (Amersham Biosciences) phosphorimager and ImageQuant software.

## Abbreviations

TEC: Ternary elongation complex; RNAP: `RNA polymerase; DNAP: DNA polymerase; Tt: Thermus thermophilus; Sc: Saccharomyces cerevisiae; Ec: Escherichia coli; Mj: Methanocaldacoccus jannachii; AMPcPP: Methylene adenosine triphosphate; RMSD: root mean square deviation.

## Competing interests

The authors declare that they have no competing interests.

## Authors’ contributions

MLK did RNAP elongation, fidelity and exonuclease III footprinting assays. KO and SAS ran molecular dynamics simulations under the direction of RIC and ZFB. CD made Sc RNAP II mutant proteins under the direction of BC. ZFB, MK and MLK wrote the paper with input from the other authors. All authors read and approved the final manuscript.

## Supplementary Material

Additional file 1 Supplementary figures and legends. Click here for file

Additional file 2 Movie M1. Closed-Mg (closed trigger loop; bonded Mg atoms) Tt RNAP TEC (*Thermus thermophilus* RNA polymerase ternary elongation complex). DNA is gold. RNA is red. ATP is red (stick). The bridge helix is green transparent. The trigger loop is green opaque. G1230 (H1), G1233 (H1), G1255 (H2), G1076 (H3), G1080 (H4) and G1081 (H4) are shown in space-filling representation. Bridge helix bending mode B1 is established and held stably through the simulation. At the beginning of the movies, closed-Mg and open RNAP TECs are very similar in conformation except for the trigger loop conformation. Click here for file

Additional file 3 Movie M2. Open (open trigger loop) Tt RNAP TEC. DNA is gold. RNA is red. ATP is red. The bridge helix is blue transparent. The trigger loop is blue opaque. G1230 (H1), G1233 (H1), G1255 (H2), G1076 (H3), G1080 (H4) and G1081 (H4) are shown in space-filling representation. Bridge helix bending mode B2 is established rapidly and held stably through the simulation. At the beginning of the movies, closed-Mg and open RNAP TECs are very similar in conformation except for the trigger loop conformation. Click here for file
