# Supplementary material for: Molecular dynamics and mutational analysis of the catalytic and translocation cycle of RNA polymerase
Source: BMC Biophys. 2012 Jun 7;5:11. doi: 10.1186/2046-1682-5-11 (PMC3533926; doi:10.1186/2046-1682-5-11)
Supplement: Additional file 1 — Supplementary figures and legends. [file 2046-1682-5-11-S1.pdf]

BMC Biophysics
Additional File 1

Molecular dynamics and mutational analysis of the catalytic and translocation cycle of RNA polymerase

Maria L Kireeva[1]
Email: kireevam@mail.nih.gov

Kristopher Opron[2]
Email: opronkri@msu.edu

Steve A Seibold[2,3,4]
Email: Seibold79@stmary.edu

Céline Domecq[5]
Email: celine.domecq@ircm.qc.ca

Robert I Cukier[3]
Email: cukier@chemistry.msu.edu

Benoit Coulombe[5,6]
Email: Benoit.Coulombe@ircm.qc.ca

Mikhail Kashlev[1]
Email: mkashlev@mail.ncifcrf.gov

Zachary F Burton[2*]
[*] Corresponding address
Email: burton@cns.msu.edu

[1] Gene Regulation and Chromosome Biology Laboratory, National Cancer Institute, Frederick, MD 21702-1201, USA

[2] Department of Biochemistry and Molecular Biology, Michigan State University, E. Lansing, MI 48824-1319, USA

[3] Department of Chemistry, Michigan State University, E. Lansing, MI 48824, USA

[4] Department of Chemistry, University of Saint Mary, Leavenworth, KS 66048, USA

[5] Gene Transcription and Proteomics Laboratory, Institut de Recherches Cliniques de Montréal (IRCM), 110, Avenue des Pins Ouest, Montréal, Québec, H2W 1R7, CANADA

[6] Department of Biochemistry, Université de Montréal, Montréal, Québec H3C 3J7, CANADA

Supplementary Movies

Additional file 2. Movie M1. Closed-Mg (closed trigger loop; bonded Mg atoms) Tt RNAP TEC (*Thermus thermophilus* RNA polymerase ternary elongation complex). DNA is gold. RNA is red. ATP is red (stick). The bridge helix is green transparent. The trigger loop is green opaque. G1230 (H1), G1233 (H1), G1255 (H2), G1076 (H3), G1080 (H4) and G1081 (H4) are shown in space-filling representation. Bridge helix bending mode B1 is established and held stably through the simulation. At the beginning of the movies, closed-Mg and open RNAP TECs are very similar in conformation except for the trigger loop conformation

Additional file 3. Movie M2. Open (open trigger loop) Tt RNAP TEC. DNA is gold. RNA is red. ATP is red. The bridge helix is blue transparent. The trigger loop is blue opaque. G1230 (H1), G1233 (H1), G1255 (H2), G1076 (H3), G1080 (H4) and G1081 (H4) are shown in space-filling representation. Bridge helix bending mode B2 is established rapidly and held stably through the simulation. At the beginning of the movies, closed-Mg and open RNAP TECs are very similar in conformation except for the trigger loop conformation.

Additional file 1.

Supplementary Figure Legends

Figure S1. **Root mean square deviation (RMSD) for closed-Mg, closed and open RNAP TEC simulations.** A) all protein Cα and nucleic C1' carbons; B) trigger loop region Cα

carbons; C) bridge helix Cα carbons; D) RNA and DNA all nitrogen and carbon atoms; E) RNA all nitrogen and carbon atoms; and F) ATP all atoms except hydrogen

Figure S2. **The closed-Mg RNAP TEC maintains near catalytic geometry during simulation.** A) Despite significant conformational changes in RNAP protein comparing closed-Mg, closed and open TEC simulations, accurate base pairing of the downstream DNA/DNA duplex and the RNA/DNA hybrid is maintained during 10.5 ns simulations. Snapshots at 10 ns are shown. B) Trajectories of the Mg-I to Mg-II (left image) and the RNA 3'-O to α-phosphate (right image) distances, indicating that the closed-Mg TEC simulation maintains near catalytic geometry while the closed and the open TEC simulations diverge

Figure S3. **Translocation of nucleic acids in simulations.** Closed-Mg (green), Closed (red) and Open (blue) simulations are compared

Figure S4. **Translocation trajectories of nucleic acids in simulations.** Translocation displacements are determined as shown in Figure 2A

Figure S5. **H1 trigger loop dynamics as a function of simulation time.** A) Δ*psi* versus simulation time for residues surrounding H1. *Phi* and *Psi* angles of amino acids define the local secondary structure of a protein. B) Secondary structure versus simulation time for residues surrounding H1. H is helix; T is turn; C is coil; G is 3_{10} helix. The images below are in secondary structure representation from 10 ns simulation snapshots. G1230 is in space-filling representation to identify H1. Blue arrows indicate hinge dynamics

Figure S6. **H2 trigger loop dynamics as a function of simulation time.** A) Δ*psi* versus simulation time for residues surrounding H2. B) Secondary structure versus simulation time for residues surrounding H2. The images below are in secondary structure representation from 10 ns simulation snapshots. G1255 is in space-filling representation to identify H2

Figure S7. **H3/H4 bridge helix dynamics as a function of simulation time.** A) Δ*psi* versus simulation time for residues surrounding H3/H4. B) Secondary structure versus simulation time for residues surrounding H3/H4. The images below are in secondary structure representation from 10 ns simulation snapshots. G1076, G1080 and G1081 are in space-filling representation to identify H3 and H4

Figure S8. **Simulation trajectories that demonstrate distinct stable conformations of the trigger loop H1 and bridge helix H3/H4 hinges in closed-Mg, closed and open TECs.** A) G1230 to T1234 Cα-carbon distance versus simulation time. This is a measure of trigger loop hinge H1 conformation and dynamics. B) G1076 to G1080 Cα-carbon distance versus simulation time. This is a measure of bridge helix hinge H3/H4 conformation and dynamics. Distances are in angstroms

Figure S9. **The competition assay for transcriptional fidelity**

Figure S10. **The competition assay compared to a conventional transcriptional fidelity assay**

Figure S11. **Switch contacts supporting closed RNAP TECs.** A) A possible ionic contact between fork residues β R420 and β D426 that develops in the closed-Mg simulation. B) A contact between β’ R1078 (mounted between the H3 and H4 bridge helix hinges) and β R428 on the fork that dissociates in the open TEC simulation but is maintained in closed and closed-Mg TECs

Figure S12. **Alternate contacts in closed and open TECs.** A) β’ Q1046 on the F-loop forms a hydrogen bond to β’ K1079 mounted between the H3 and H4 bridge helix hinges in the closed-Mg TEC but not the open TEC. B) β’ R1078 on the bridge helix can form a hydrogen bond to the backbone oxygen of β D426 on the fork

Figure S13. **Alternate contacts in closed and open TECs.** A) β’ K1079 on the bridge helix forms an ionic contact to β D429 on the fork in the open TEC but not the closed TECs. B) β’ R1078 on the bridge helix forms a hydrogen bond to β V427 on the fork in the open TEC but not the closed TECs

Figure S14. **Alternate contacts in closed and open TECs.** Switch residue β’ D784 forms a hydrogen bond to β E686 in the closed TECs but not the open TEC. The stair step pattern observed in the closed TEC simulation results from rotation of the β’ D784 carboxyl group

Figure S15. **Alternate contacts in closed and open TECs.** A) Switch residue β’ D784 forms a hydrogen bond to β N683 in the closed-mg TEC but not the closed or the open TEC. B) The

switch residue β' D784 backbone oxygen forms a hydrogen bond to β' S964 in the closed TECs but not the open TEC

Figure S16. **Alternate contacts in closed and open TECs.** A) The switch residue β' D784 carboxyl group forms a hydrogen bond to β' S964 in the open TEC but not the closed TECs. Contacts to β' D784 OD1 and OD2 are shown to indicate rotation of the β' D784 carboxyl group. B) The switch residue β' D784 carboxyl group forms a hydrogen bond to β N872

Figure S17. **Alternate contacts in closed and open TECs.** A) The switch residue β D685 forms an ionic contact to β R557 in the open TEC but not the closed TECs

Figure S18. **Water counts within 4 angstroms of A) ATP N3 and B) RNA 3'-O**

Figure S19. **One water molecule stably associates with Mg-I in the closed-Mg RNAP TEC simulation.** Left panel) Water molecule "A" interacts strongly with Mg-I. Right panel) Water "A" interacting with Mg-I

Figure S20. **Three water molecules stably associate with Mg-I in the open RNAP TEC simulation.** Left panel) Water molecules (A, B and C) interact strongly with Mg-I. Right panel) Water molecules interact with Mg-I

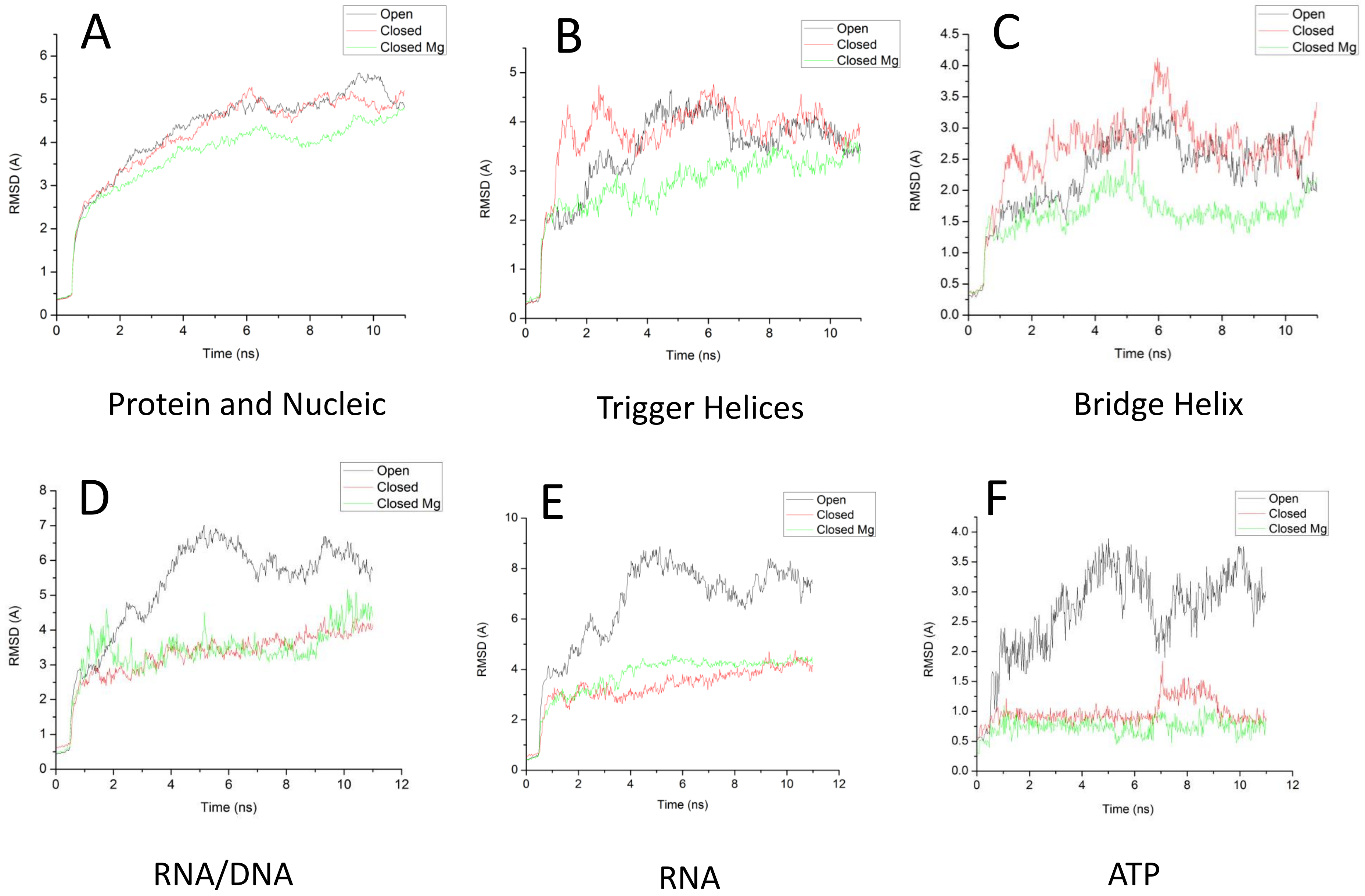
A
Open
Closed
Closed Mg
RMSD (A)
Time (ns)
Protein and Nucleic
B
Open
Closed
Closed Mg
RMSD (A)
Time (ns)
Trigger Helices
C
Open
Closed
Closed Mg
RMSD (A)
Time (ns)
Bridge Helix
D
Open
Closed
Closed Mg
RMSD (A)
Time (ns)
RNA/DNA
E
Open
Closed
Closed Mg
RMSD (A)
Time (ns)
RNA
F
Open
Closed
Closed Mg
RMSD (A)
Time (ns)
ATP

S1

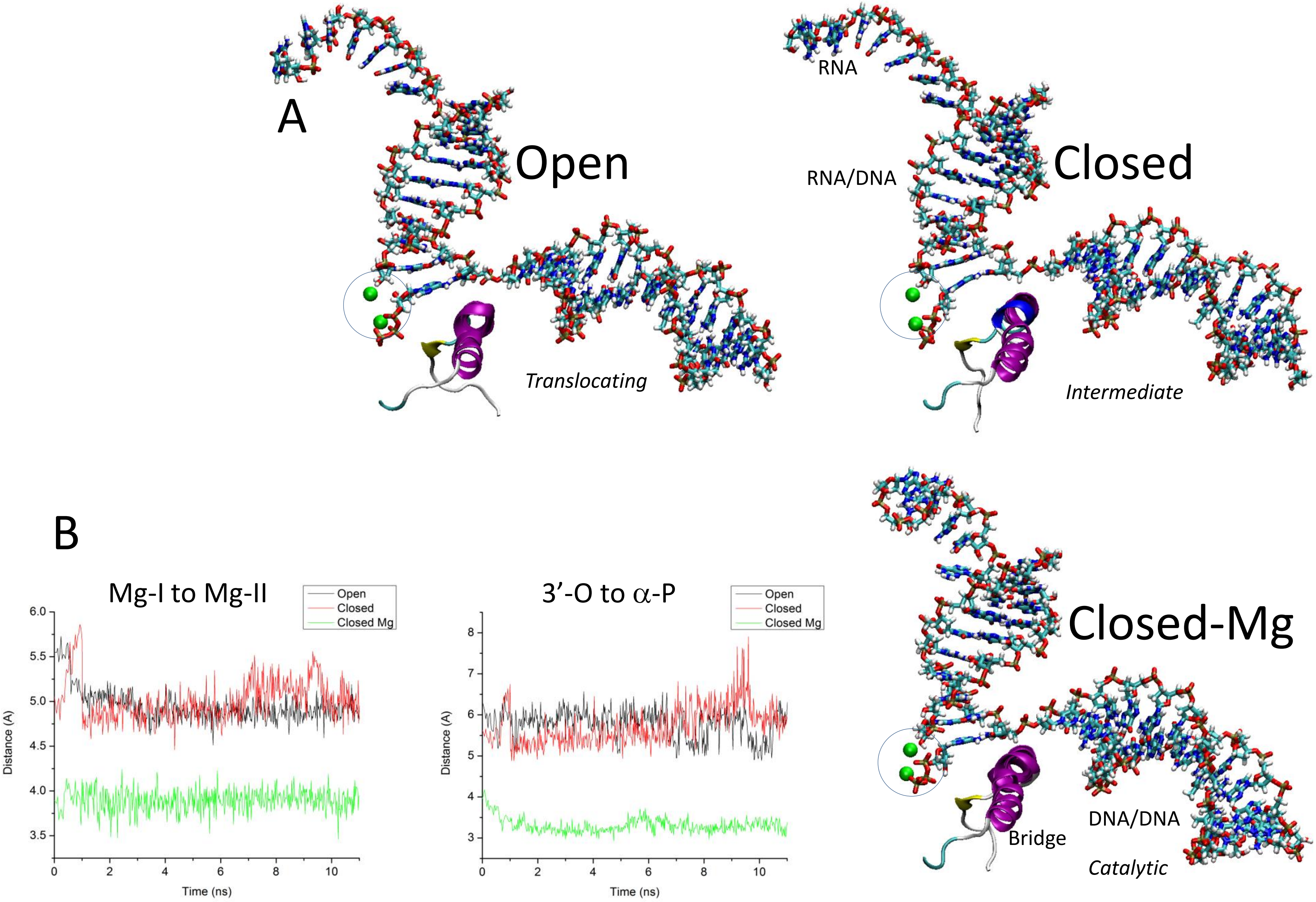
A
RNA
RNA/DNA
Open
Closed
Translocating
Intermediate
B
Mg-I to Mg-II
3'-O to α-P
Open
Closed
Closed Mg
Distance (A)
Time (ns)
Closed-Mg
DNA/DNA
Bridge
Catalytic

S2

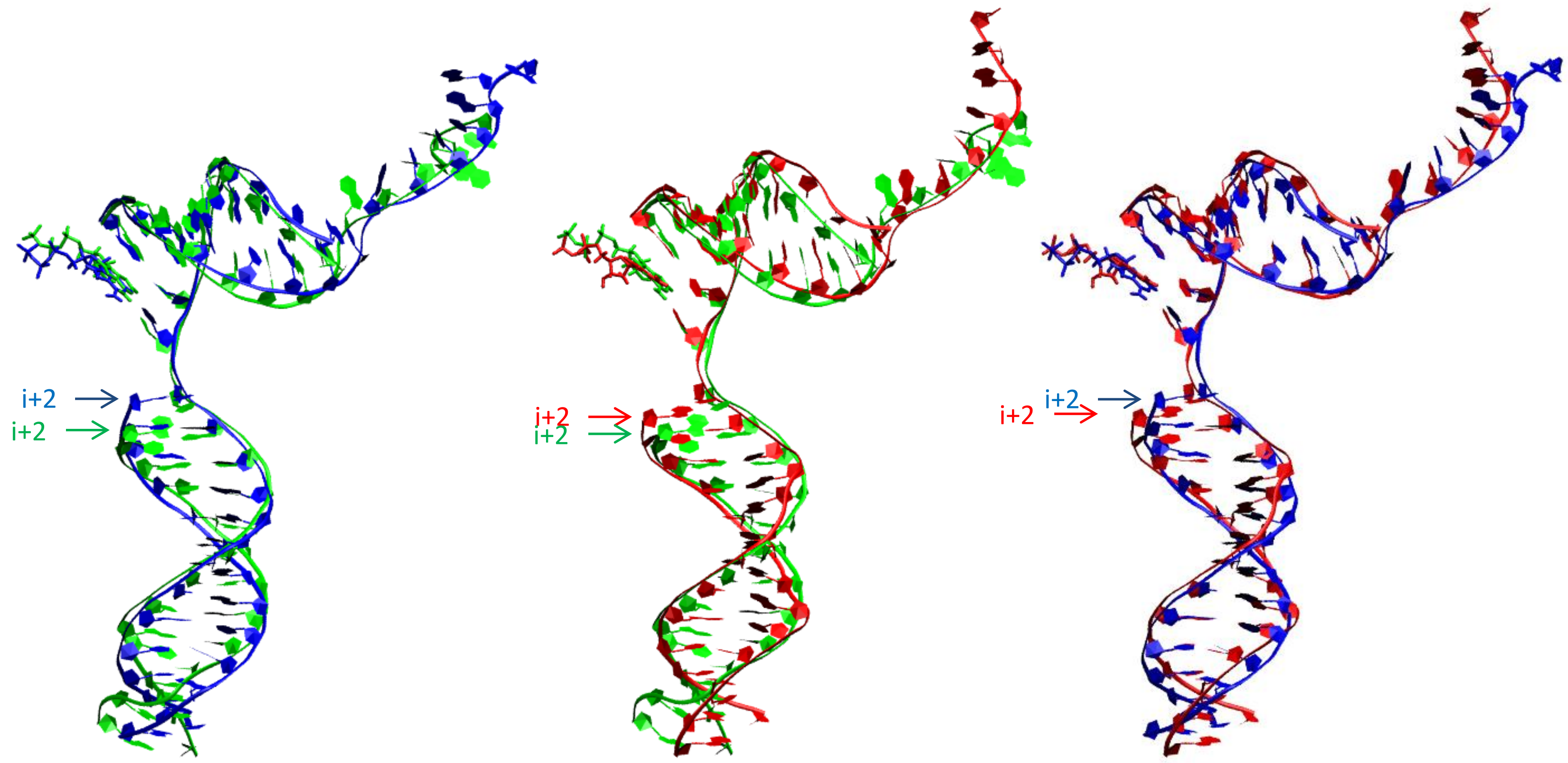

Closed-Mg vs. Open

Closed-Mg vs. Closed

Closed vs. Open

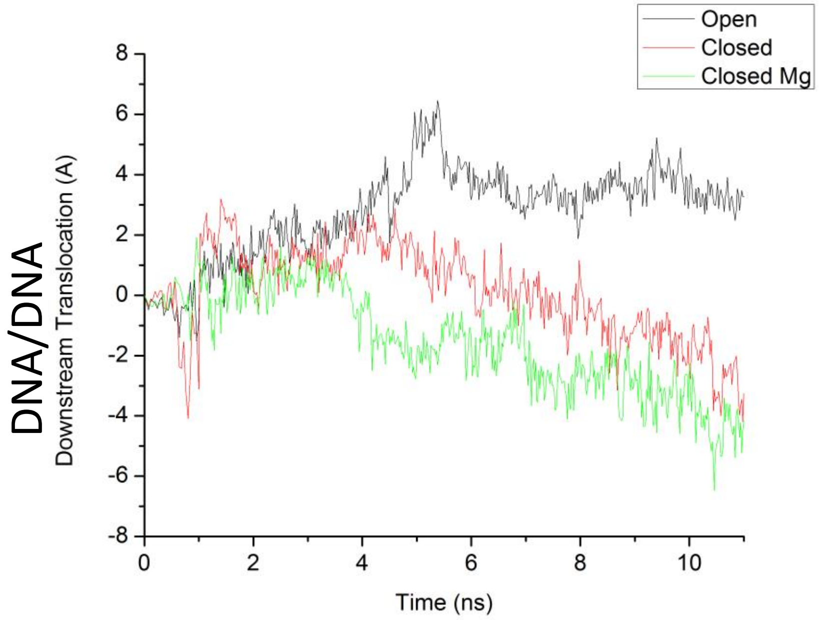
DNA/DNA
Downstream Translocation (A)
Time (ns)
Open
Closed
Closed Mg
8
6
4
2
0
-2
-4
-6
-8
0
2
4
6
8
10

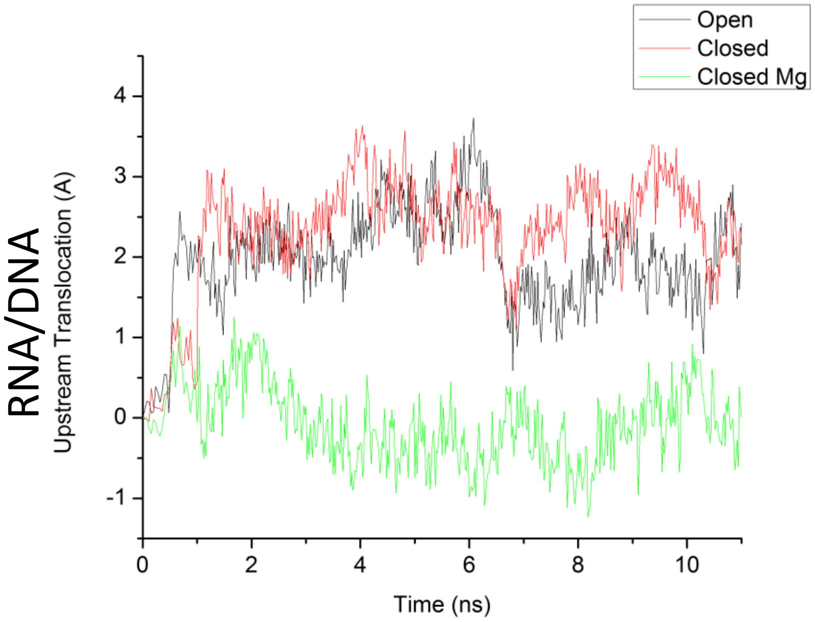
RNA/DNA
Upstream Translocation (A)
Time (ns)
Open
Closed
Closed Mg
4
3
2
1
0
-1
0
2
4
6
8
10

S4

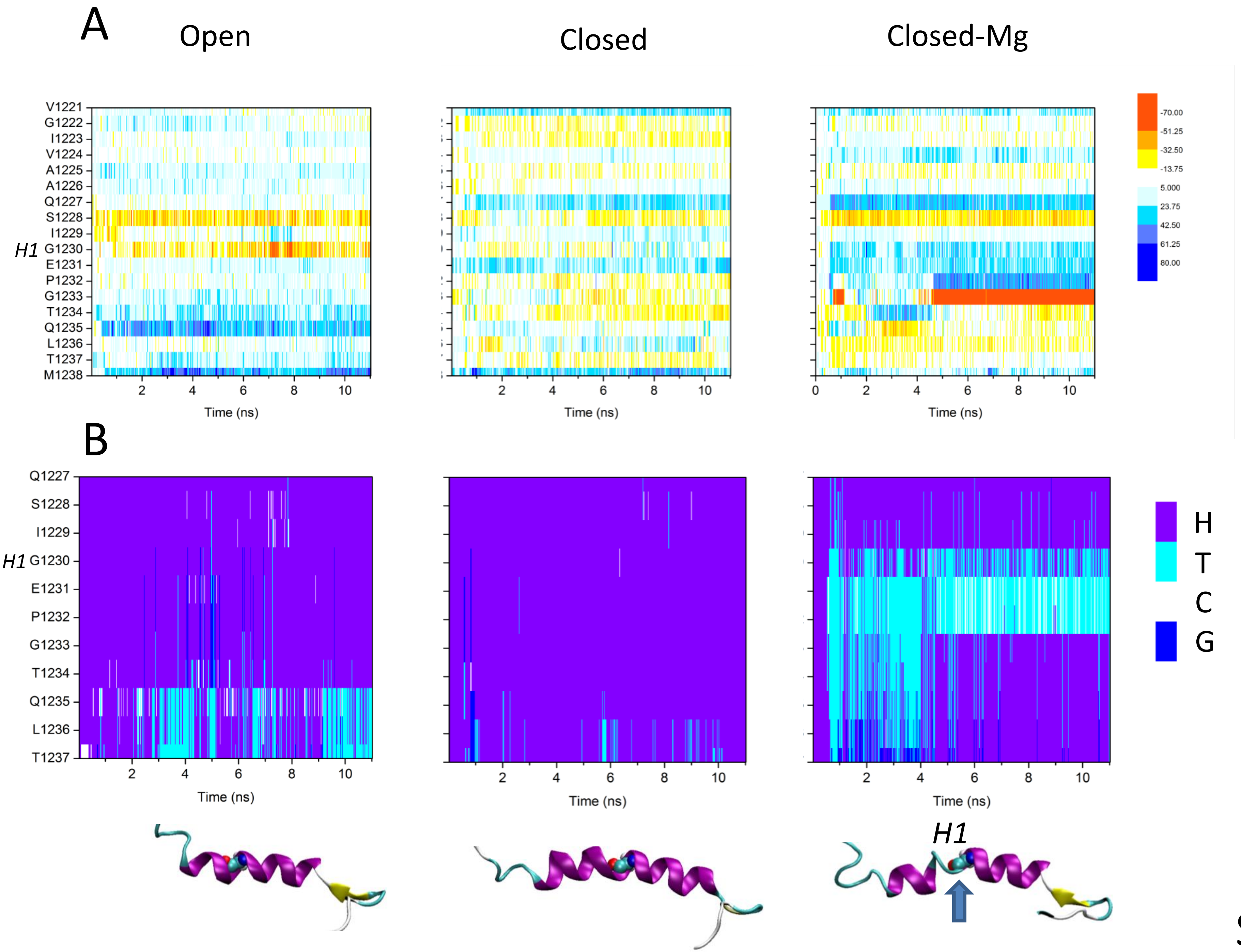

A
Open
Closed
Closed-Mg
V1221
G1222
I1223
V1224
A1225
A1226
Q1227
S1228
I1229
H1 G1230
E1231
P1232
G1233
T1234
Q1235
L1236
T1237
M1238
Time (ns)
-70.00
-51.25
-32.50
-13.75
5.000
23.75
42.50
61.25
80.00
B
H
T
C
G
H1

S5

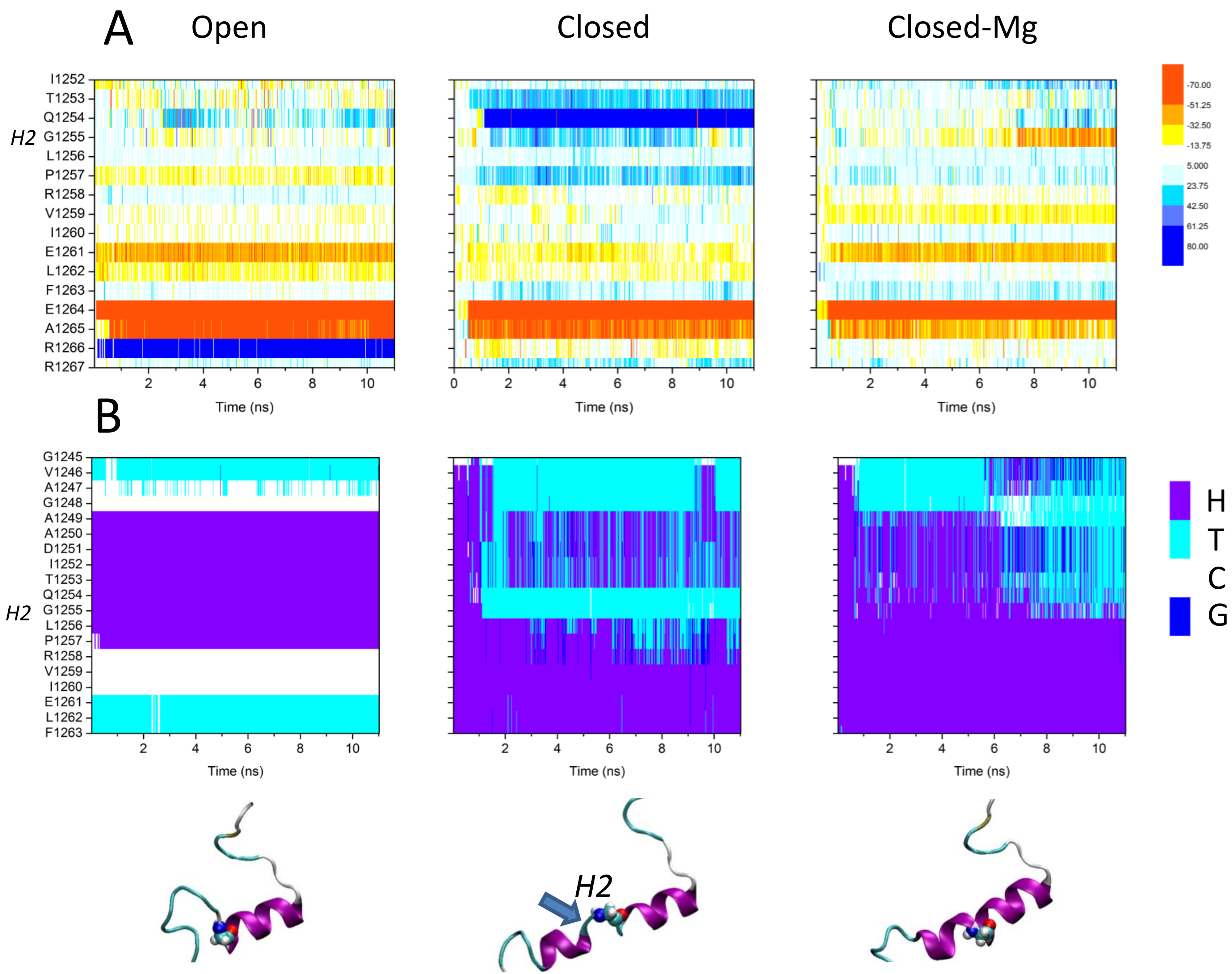
A
Open
Closed
Closed-Mg
I1252
T1253
Q1254
H2
G1255
L1256
P1257
R1258
V1259
I1260
E1261
L1262
F1263
E1264
A1265
R1266
R1267
Time (ns)
-70.00
-51.25
-32.50
-13.75
5.000
23.75
42.50
61.25
80.00
B
G1245
V1246
A1247
G1248
A1249
A1250
D1251
H
T
C
G

S6

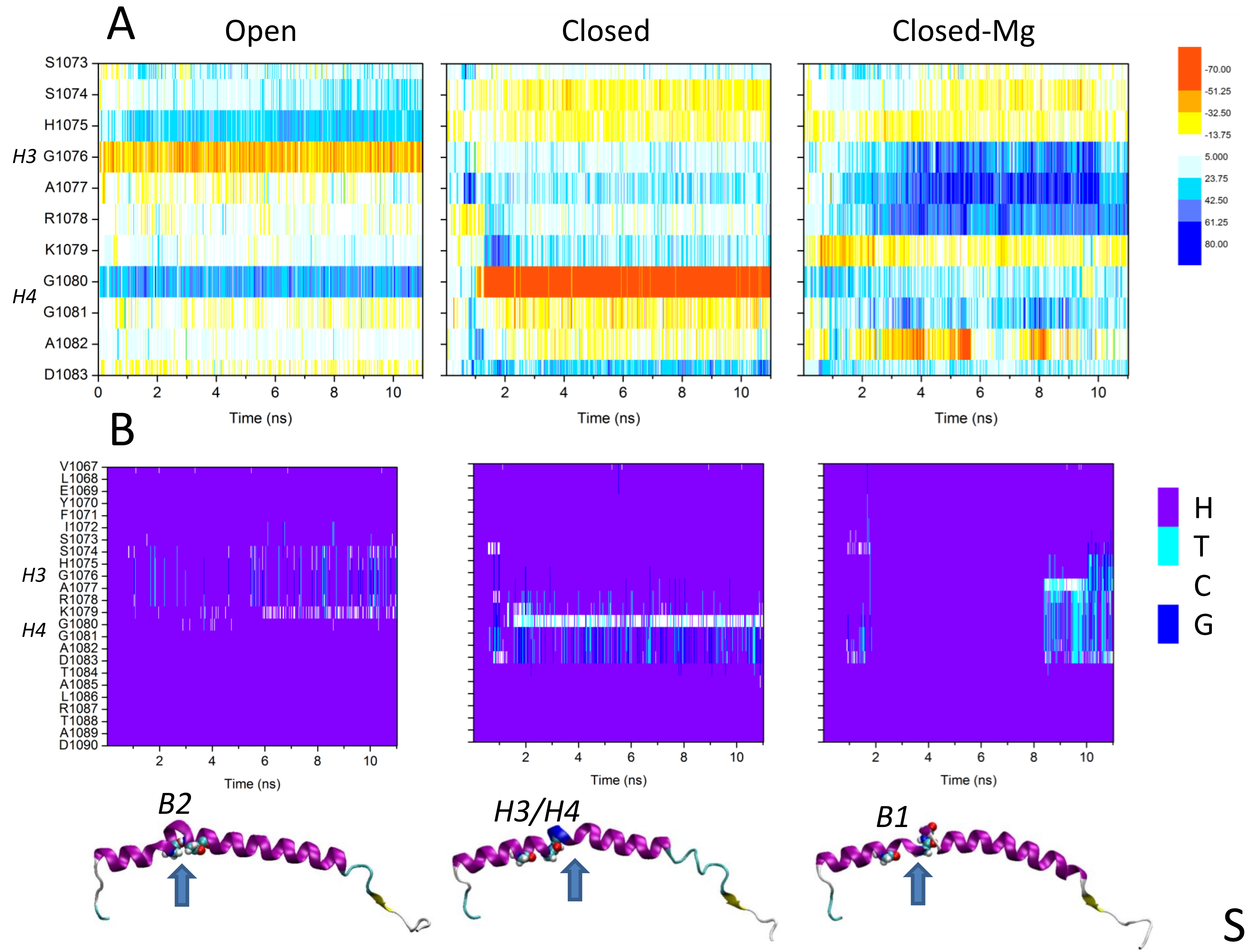
A
Open
Closed
Closed-Mg
S1073
S1074
H1075
H3 G1076
A1077
R1078
K1079
G1080
H4
G1081
A1082
D1083
-70.00
-51.25
-32.50
-13.75
5.000
23.75
42.50
61.25
80.00
Time (ns)
B
V1067
L1068
E1069
Y1070
F1071
I1072
S1073
S1074
H1075
H3 G1076
A1077
R1078
K1079
H4 G1080
G1081
A1082
D1083
T1084
A1085
L1086
R1087
T1088
A1089
D1090
H
T
C
G
Time (ns)
B2
H3/H4
B1

S7

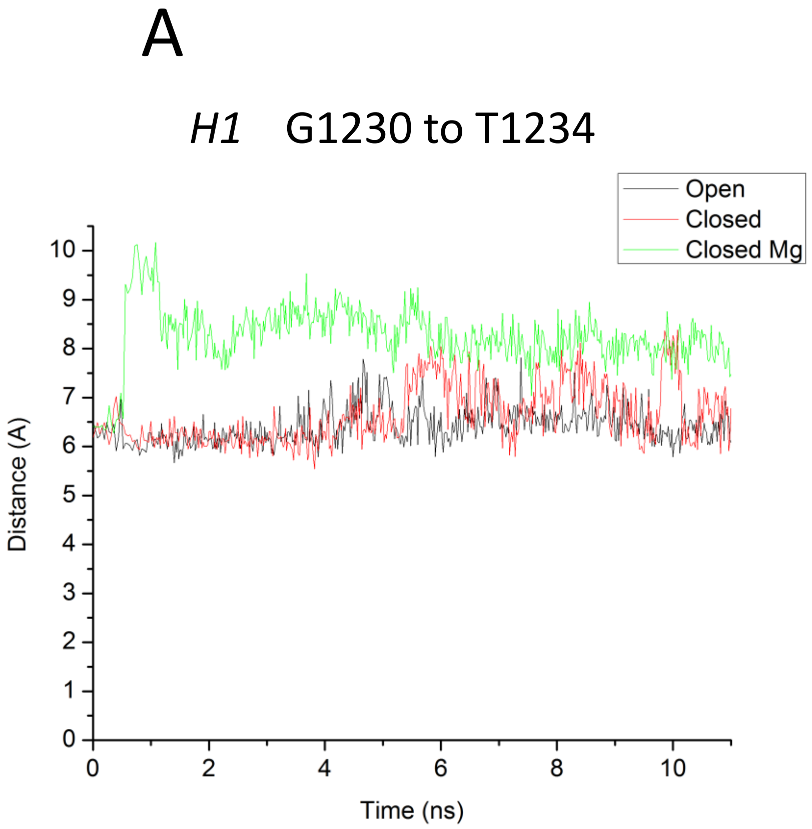
A
H1 G1230 to T1234
Open
Closed
Closed Mg
Distance (A)
Time (ns)
0
1
2
3
4
5
6
7
8
9
10
0
2
4
6
8
10

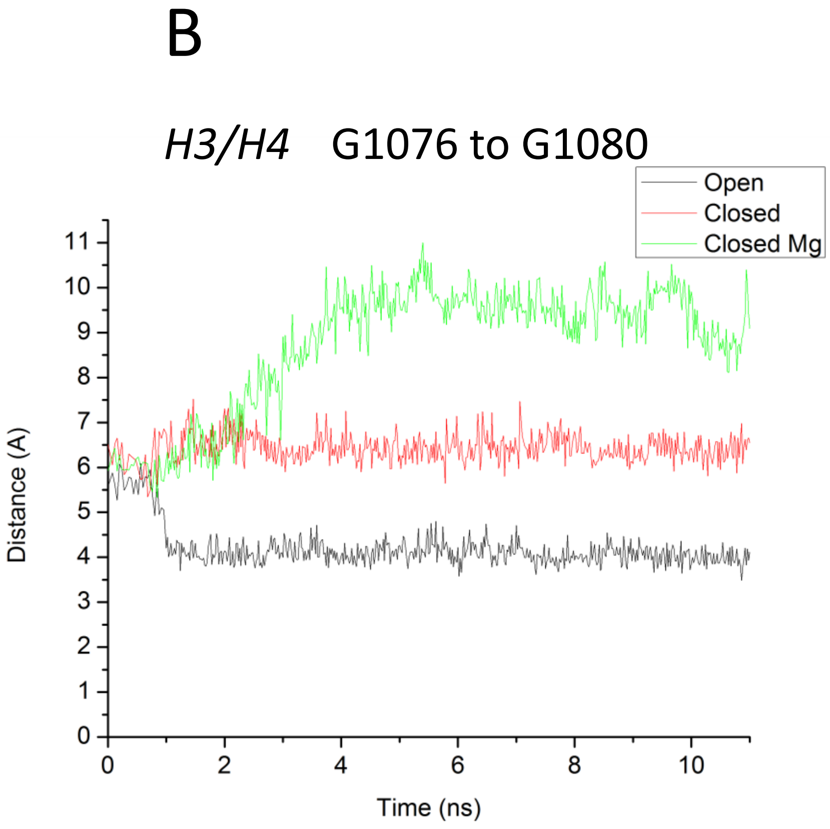
B
H3/H4 G1076 to G1080
Open
Closed
Closed Mg
Distance (A)
Time (ns)
0
1
2
3
4
5
6
7
8
9
10
11
0
2
4
6
8
10

The “competition” assay for transcription fidelity

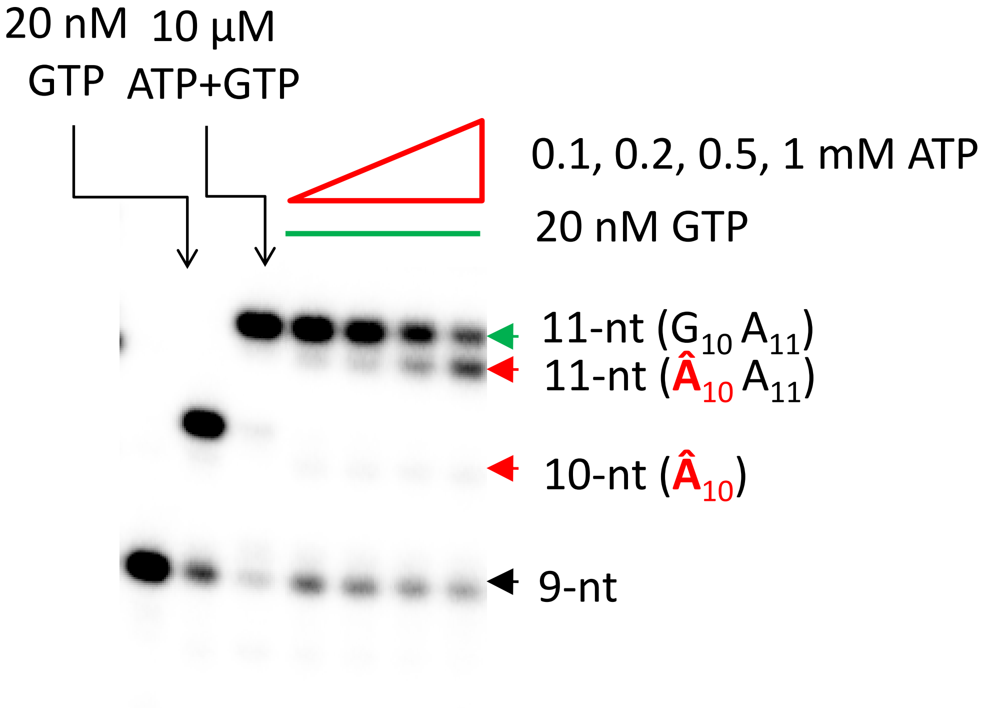

$$\textbf{Fidelity} = \frac{\textbf{Correct product (\%)}}{\textbf{Mismatched products (\%)}} \times \frac{\textbf{[non-cognate NTP]}}{\textbf{[cognate NTP]}}$$

WT RNAP II fidelity = $(6.8 \pm 0.1) \times 10^4$

Competition assay and the conventional approach to transcription fidelity measurement produce similar results

Conventional assay:

- The incorporation rates for the cognate and non-cognate NMPs are determined at different [NTP]

- The data are fitted with an equation
$k = k_{pol} \times [NTP]/(K_d + [NTP])$
and the *apparent* k_{pol} and K_d are determined

- Fidelity is calculated as the ratio of k_{pol}/K_d parameters for the cognate and non-cognate substrates

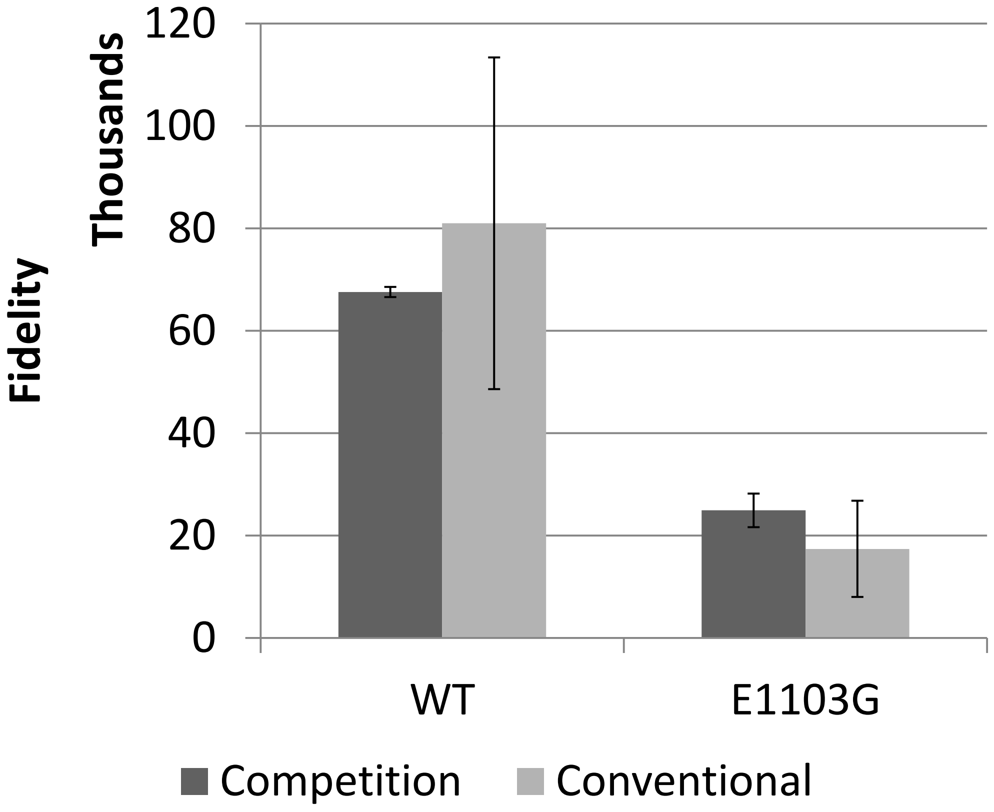

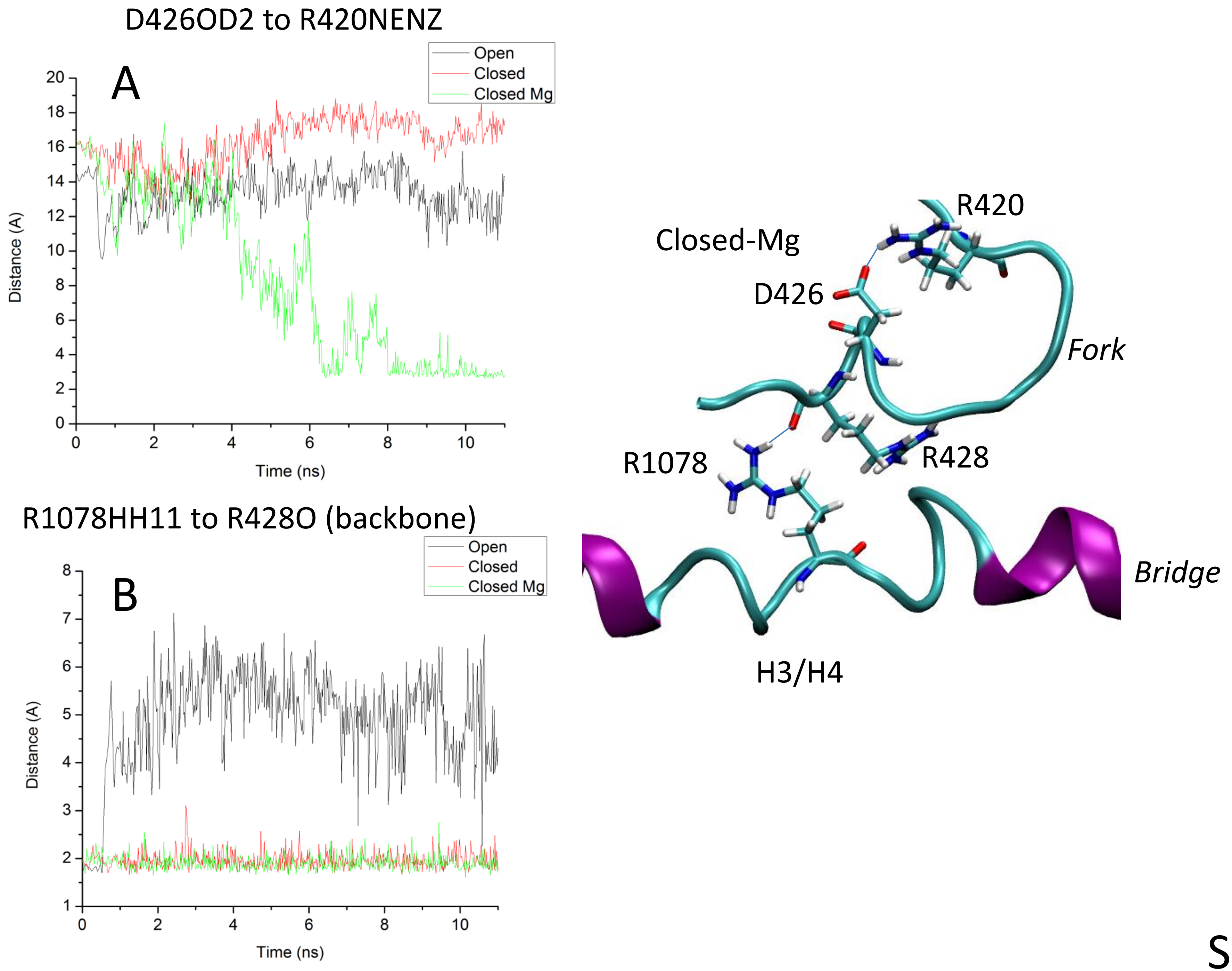

D426OD2 to R420NENZ
A
Open
Closed
Closed Mg
Distance (A)
Time (ns)
R1078HH11 to R428O (backbone)
B
Open
Closed
Closed Mg
Distance (A)
Time (ns)
Closed-Mg
R420
D426
Fork
R1078
R428
Bridge
H3/H4

S11

Q1046OE1 to K1079HZ1

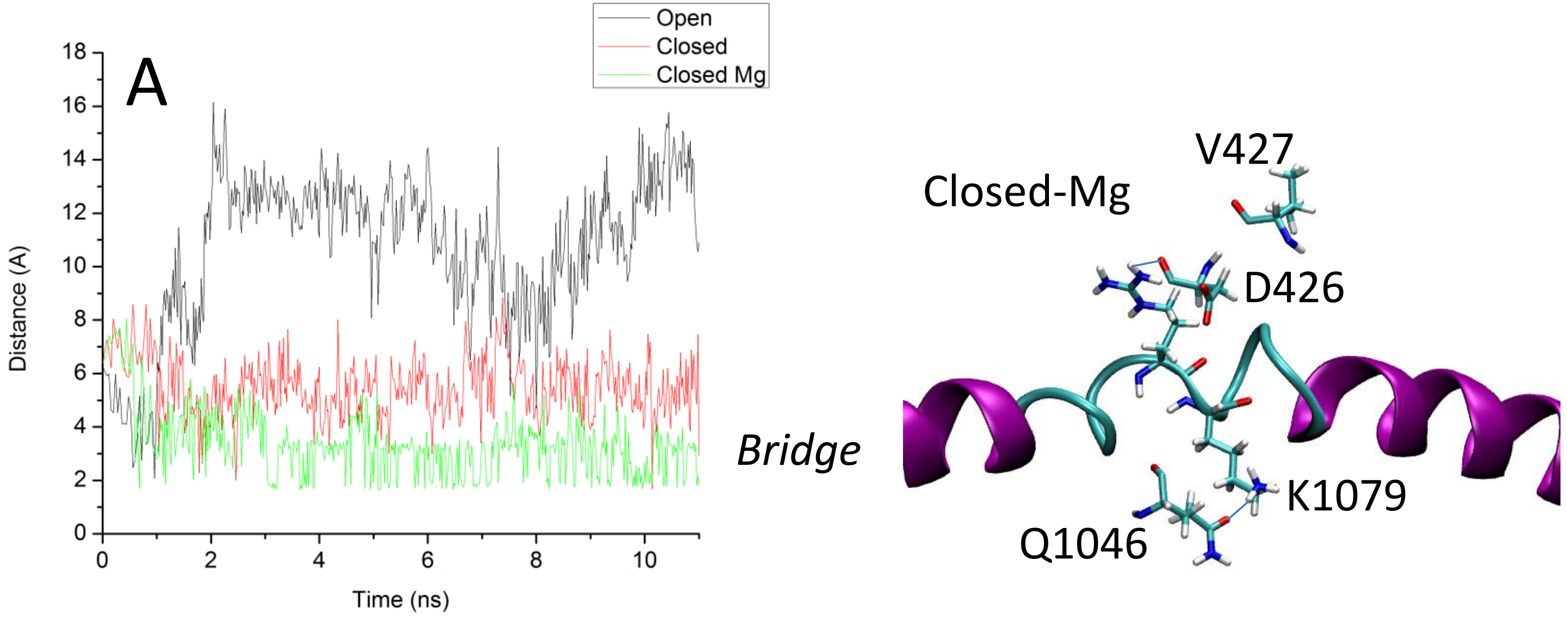

R1078HH1 to D426O backbone

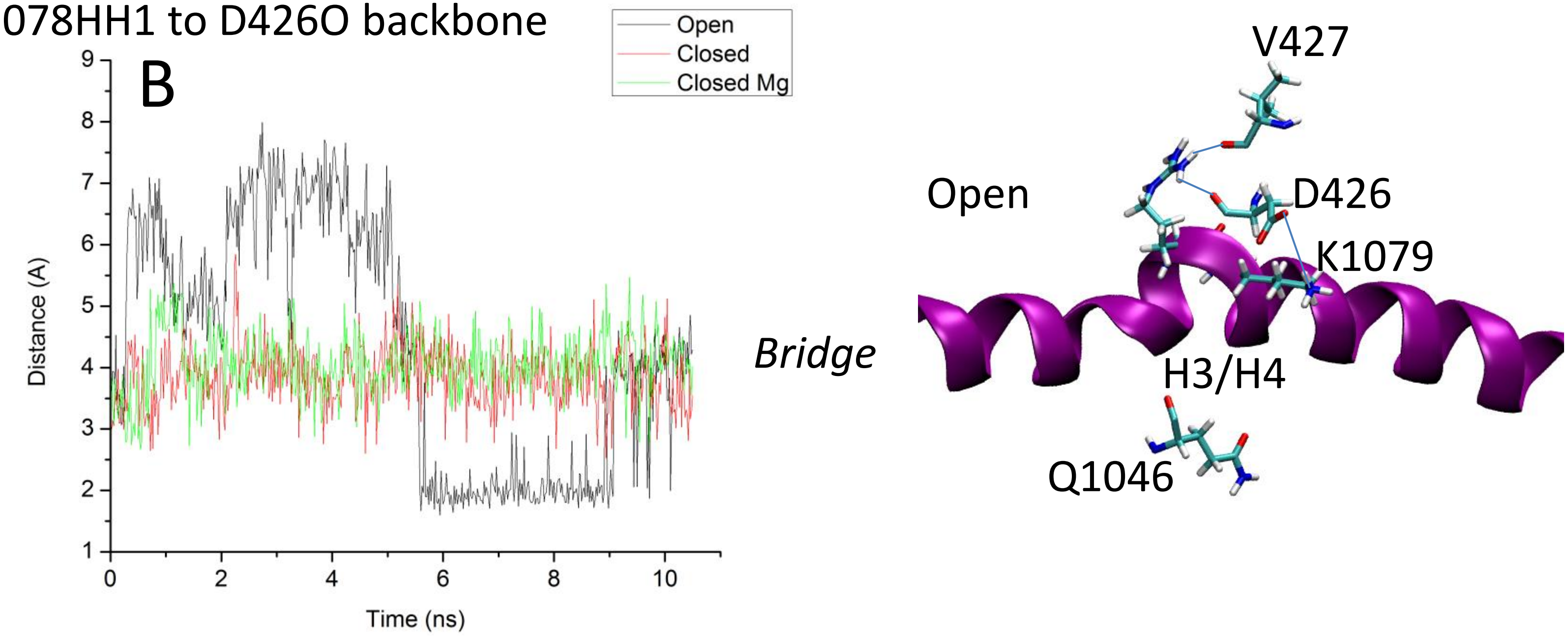

S12

D429OD2 to K1079H23H

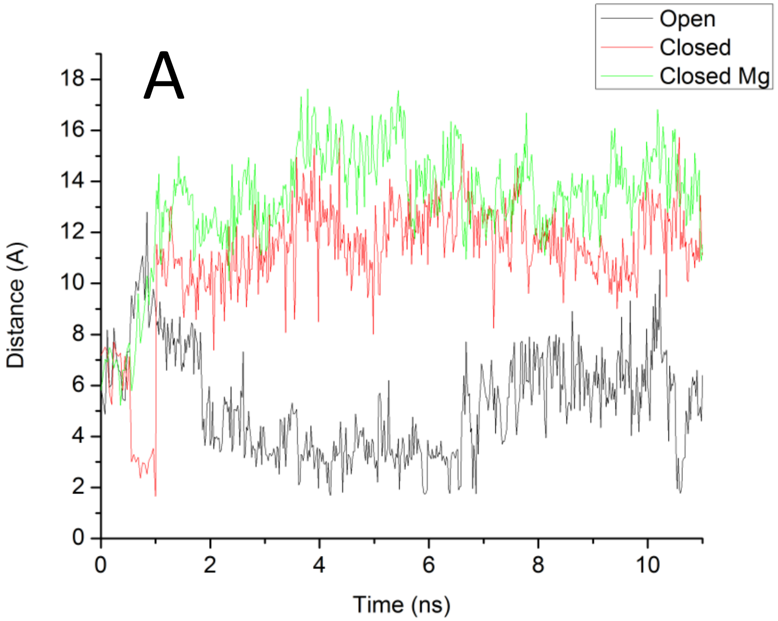

R1078HH12 to V427O

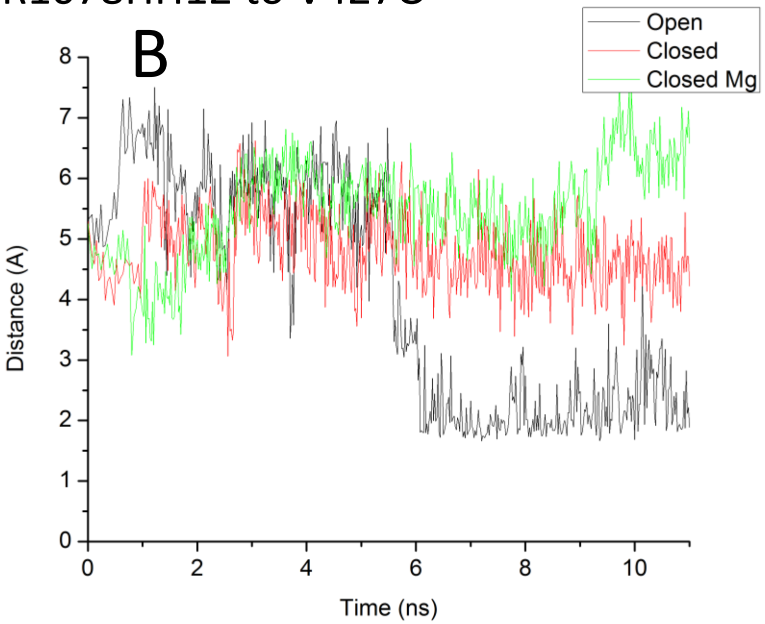

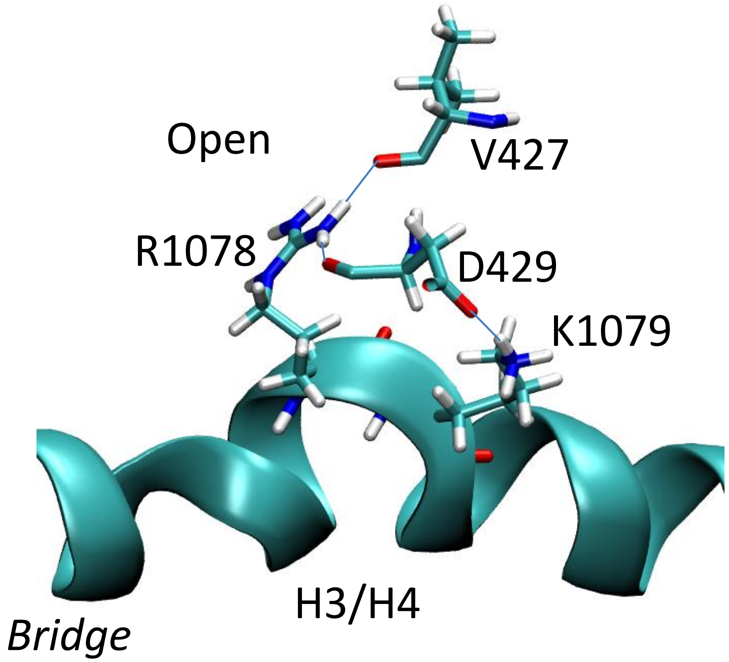

D784OD1 to E686H (backbone)

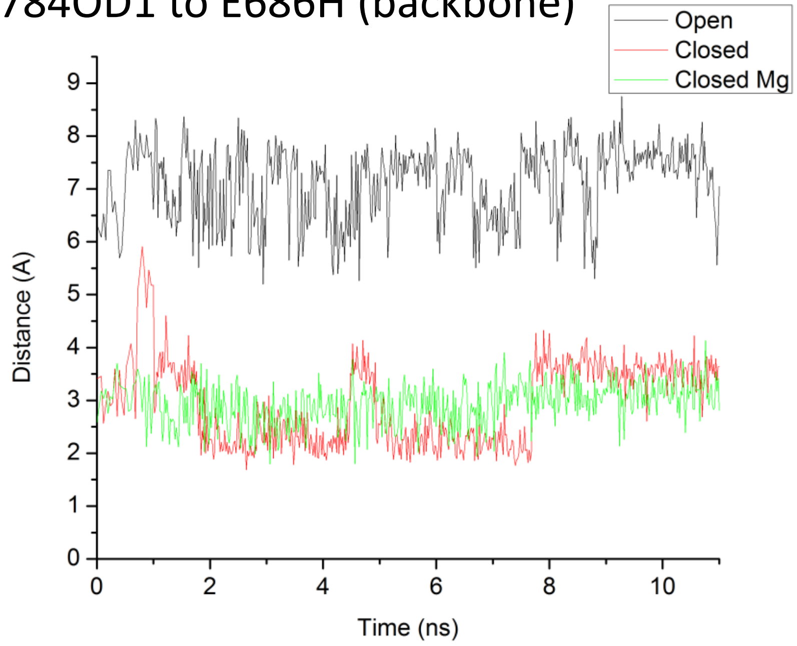

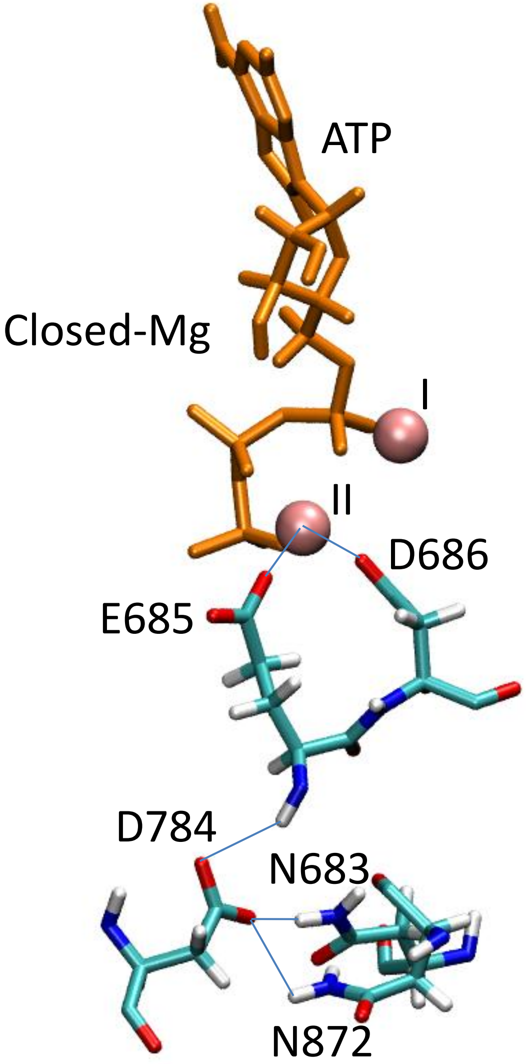

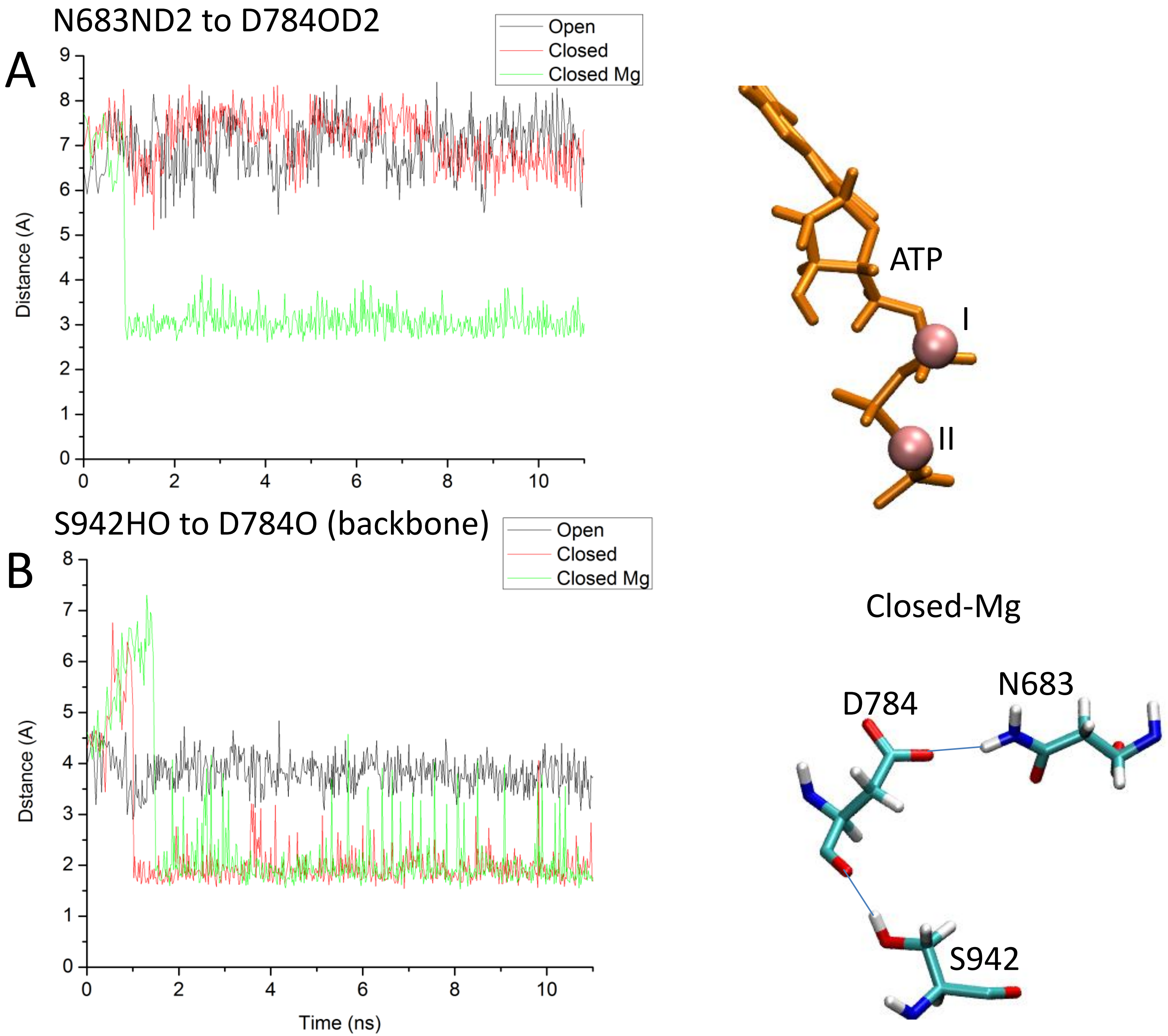

N683ND2 to D784OD2
A
Open
Closed
Closed Mg
Distance (A)
ATP
I
II
S942HO to D784O (backbone)
B
Open
Closed
Closed Mg
Dstance (A)
Time (ns)
Closed-Mg
D784
N683
S942

S15

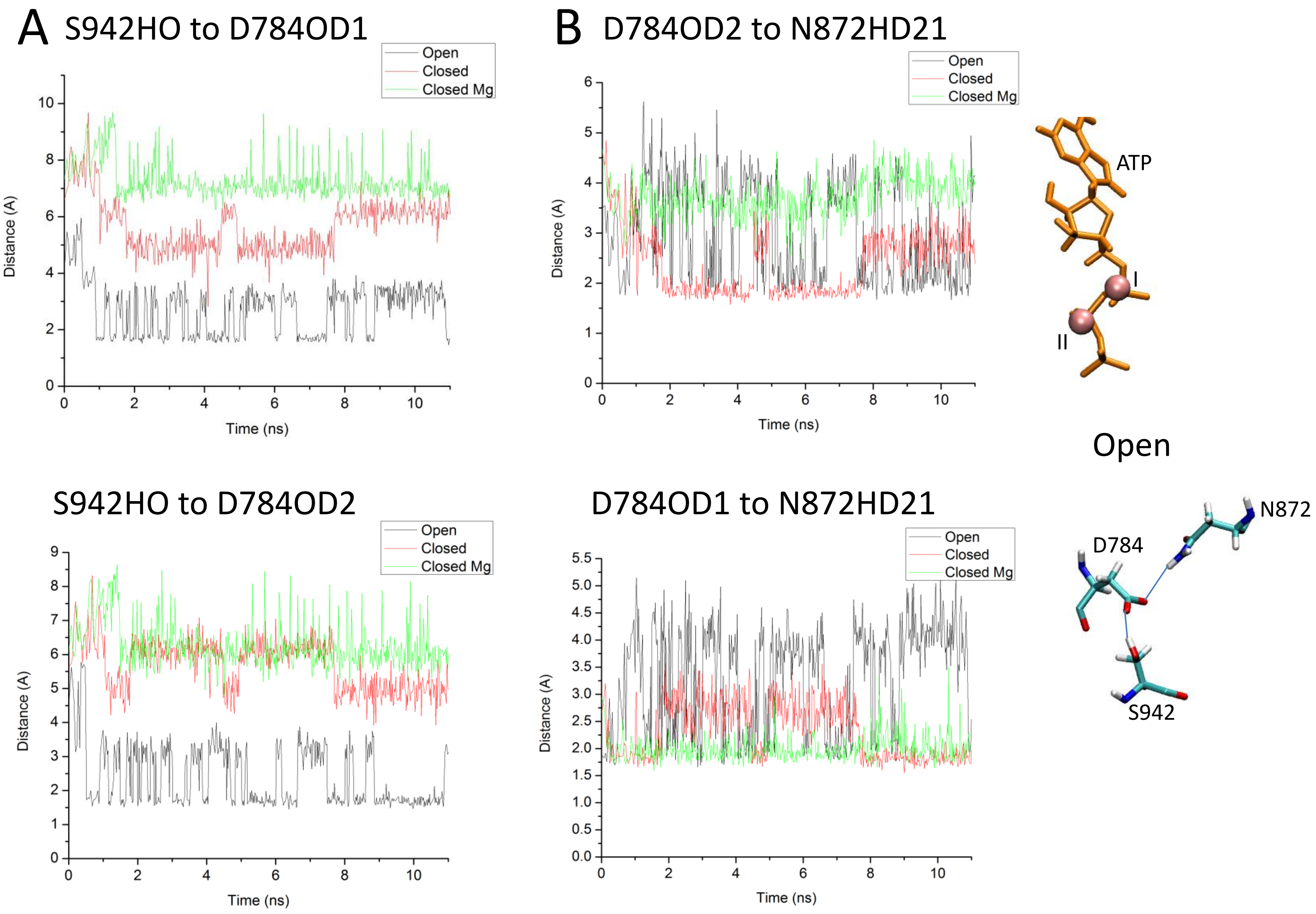

A
S942HO to D784OD1
Open
Closed
Closed Mg
Distance (A)
Time (ns)
B
D784OD2 to N872HD21
Open
Closed
Closed Mg
Distance (A)
Time (ns)
ATP
I
II
Open
S942HO to D784OD2
Open
Closed
Closed Mg
Distance (A)
Time (ns)
D784OD1 to N872HD21
Open
Closed
Closed Mg
Distance (A)
Time (ns)
N872
D784
S942

D685OD2 to R557HH22

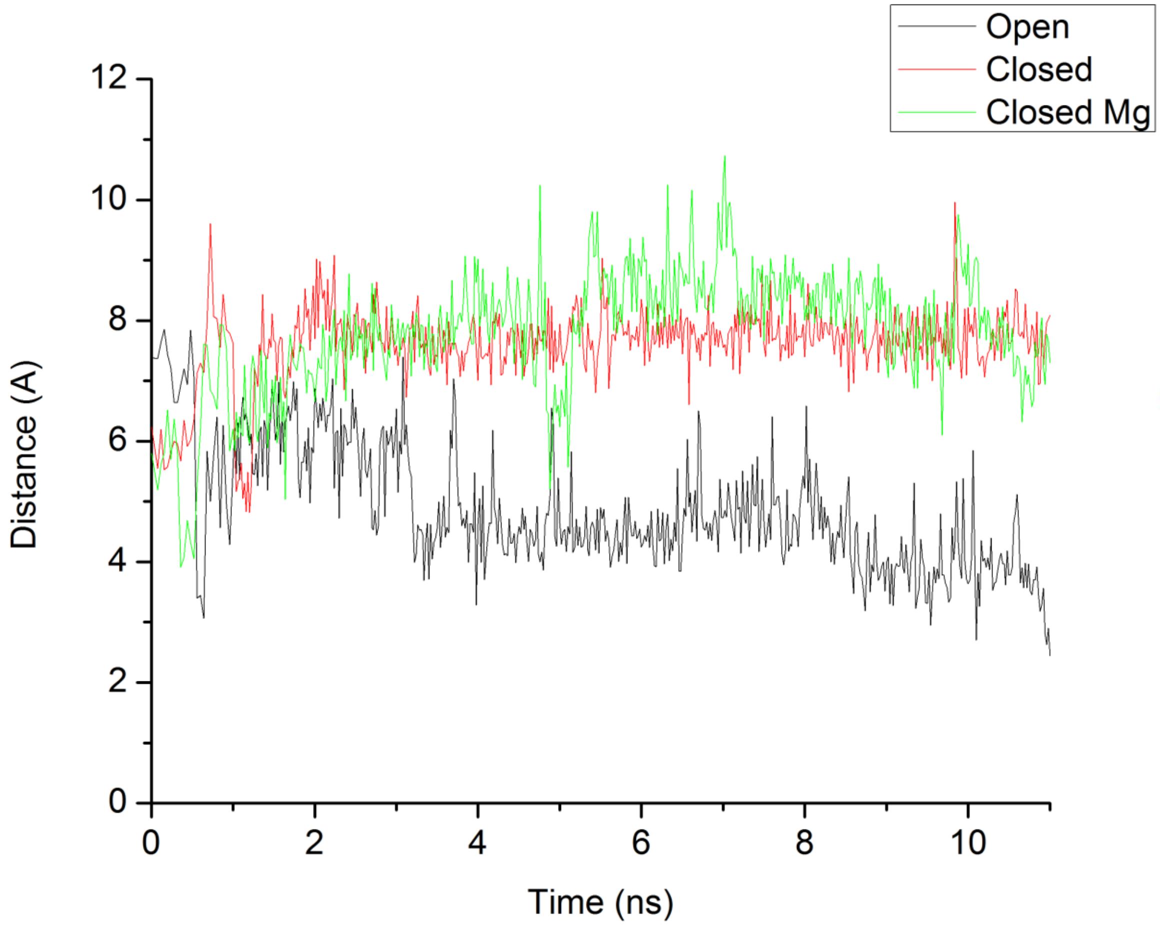

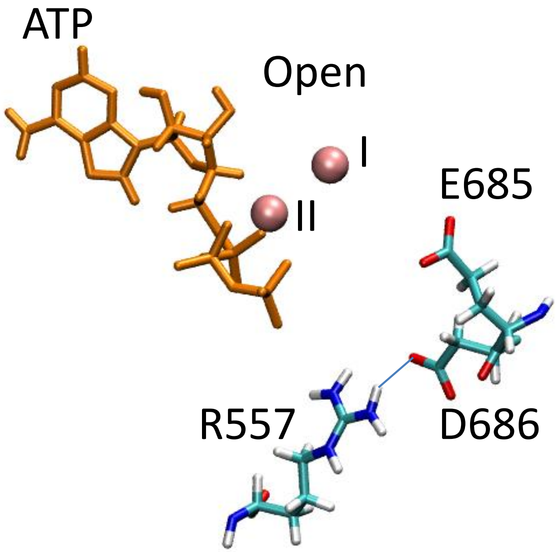

A ATP N3

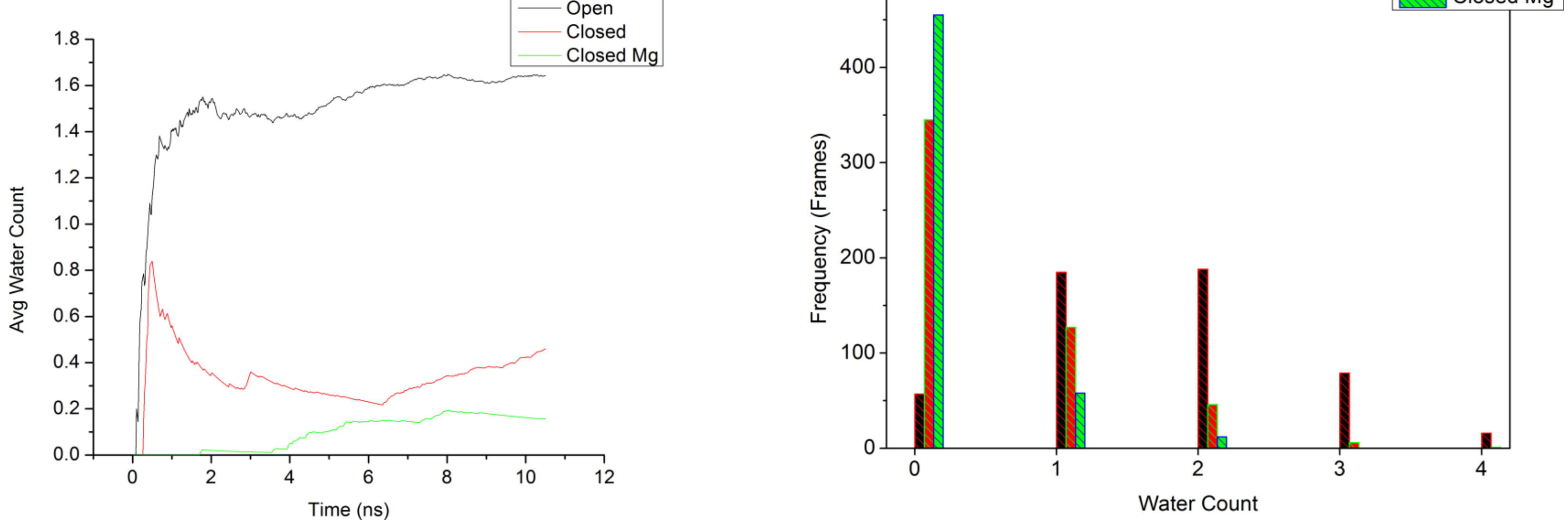

B RNA 3'O

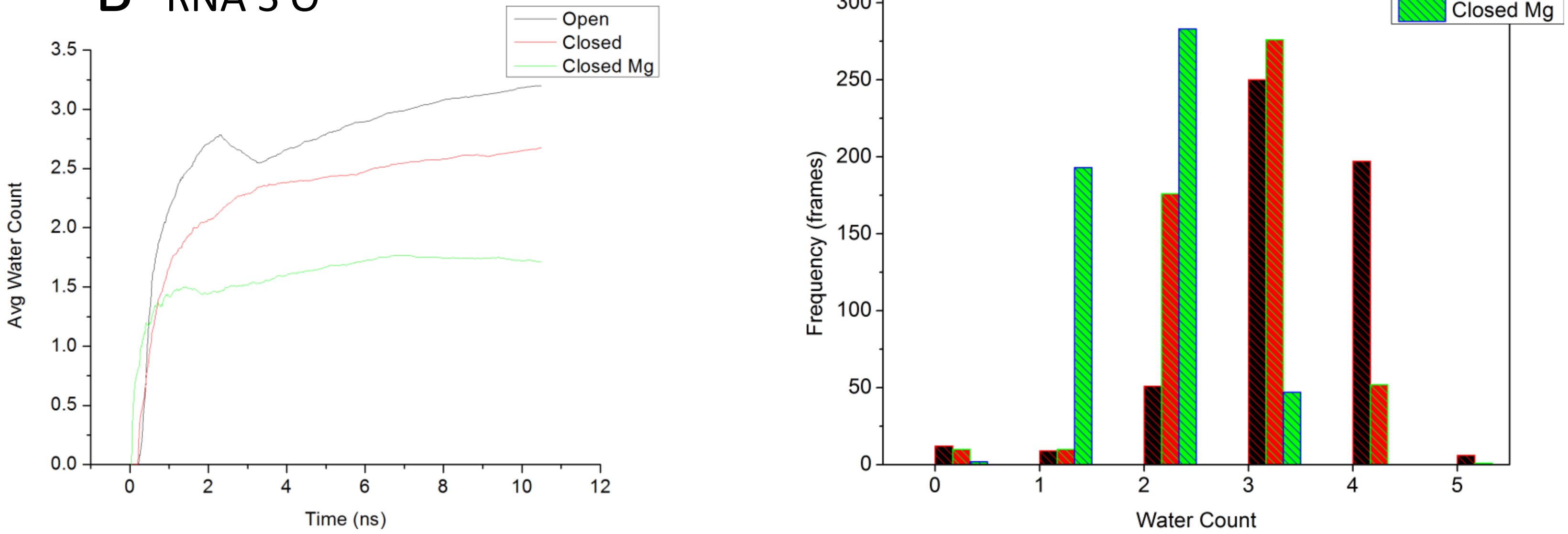

Closed-Mg RNA 3’-O

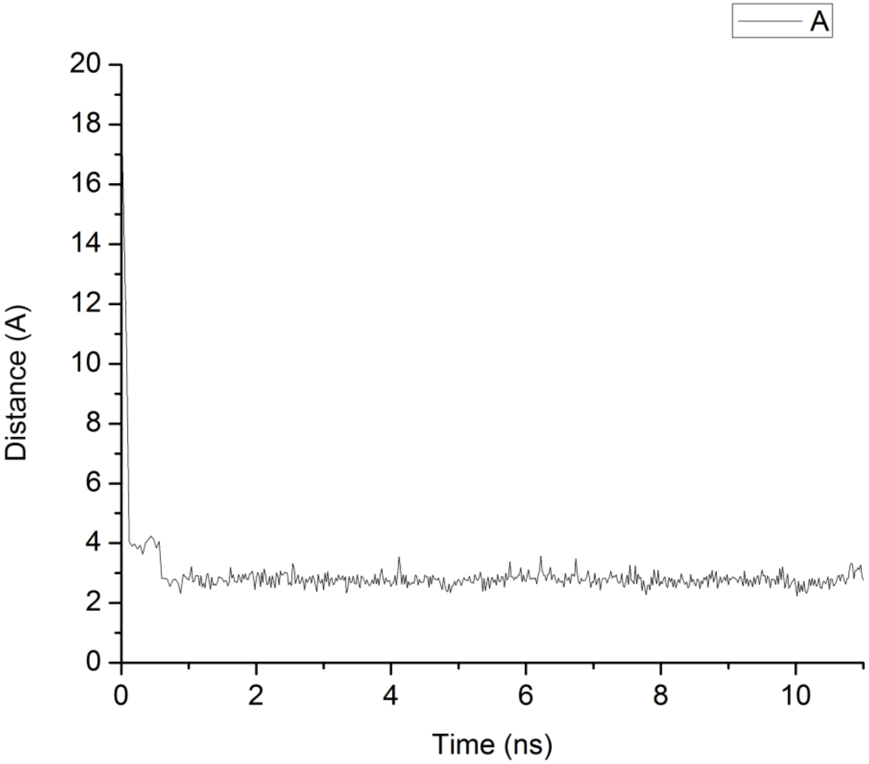

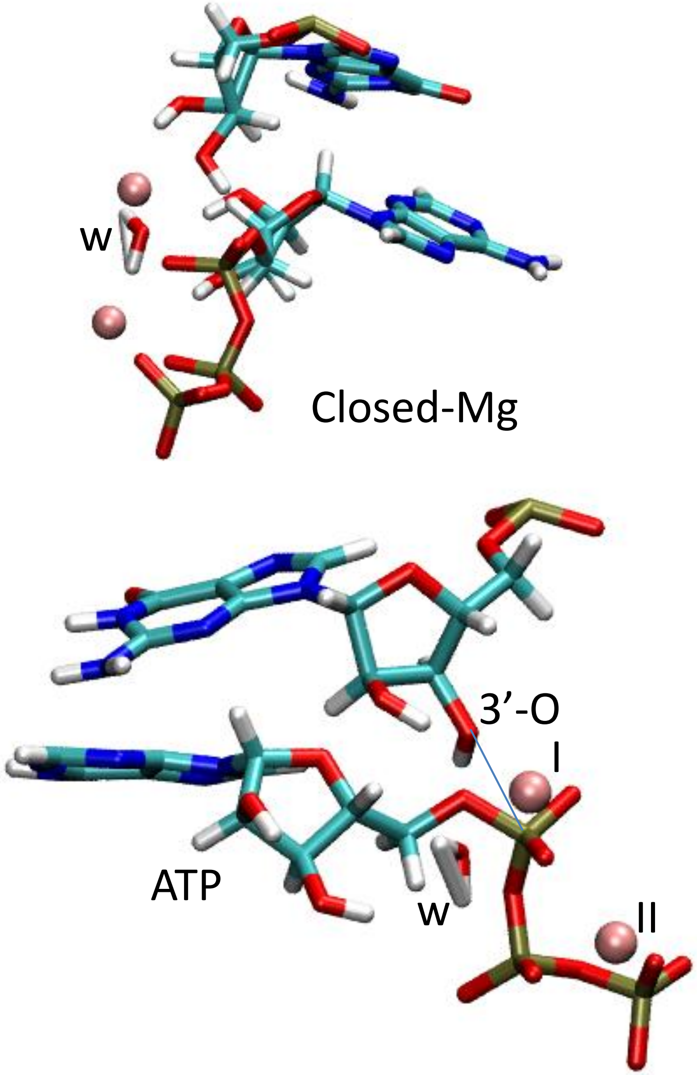

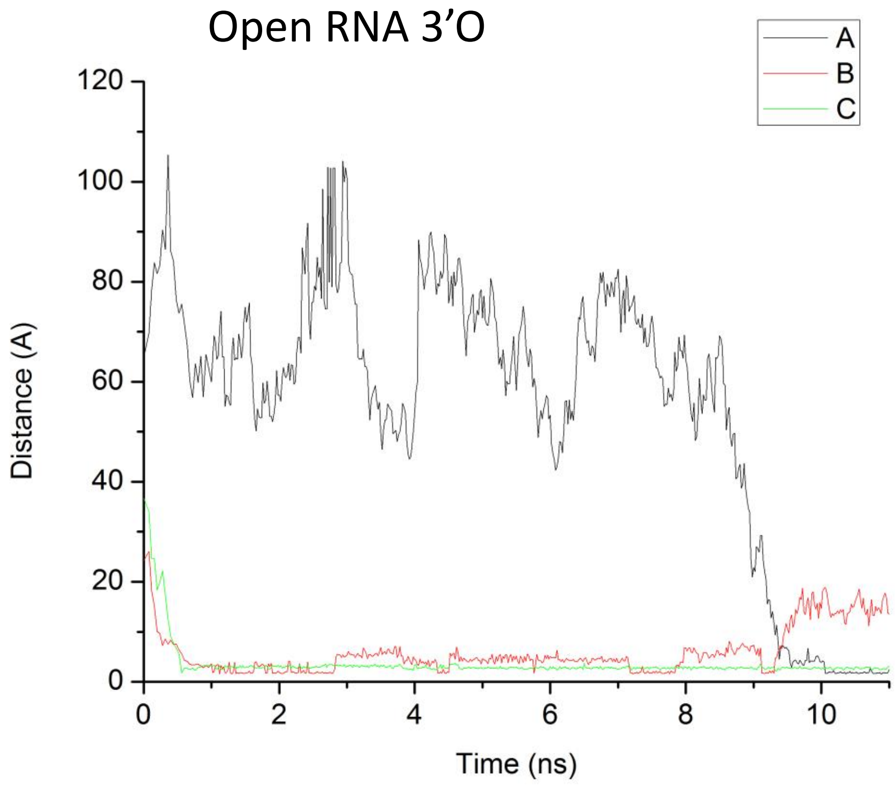
Open RNA 3’O
A
B
C
120
100
80
60
40
20
0
Distance (A)
0
2
4
6
8
10
Time (ns)

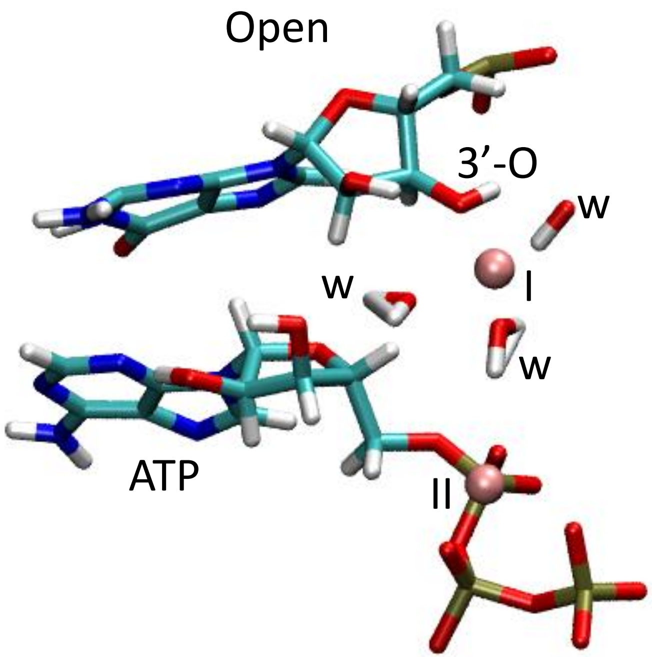
Open
3’-O
w
w
I
w
ATP
II
